# Metabolic network analysis of pre-ASD newborns and 5-year-old children with autism spectrum disorder

**DOI:** 10.1038/s42003-024-06102-y

**Published:** 2024-05-10

**Authors:** Sai Sachin Lingampelly, Jane C. Naviaux, Luke S. Heuer, Jonathan M. Monk, Kefeng Li, Lin Wang, Lori Haapanen, Chelsea A. Kelland, Judy Van de Water, Robert K. Naviaux

**Affiliations:** 1 The Mitochondrial and Metabolic Disease Center, University of California, San Diego School of Medicine, San Diego, CA 92103-8467 USA; 2 Department of Medicine, University of California, San Diego School of Medicine, San Diego, CA 92103-8467 USA; 3 Department of Neuroscience, University of California, San Diego School of Medicine, San Diego, CA 92103-8467 USA; 4 The UC Davis MIND Institute, University of California, Davis, Davis, CA 95616 USA; 5 Department of Rheumatology and Allergy, School of Veterinary Medicine, University of California, Davis, Davis, CA 95616 USA; 6 Department of Pediatrics, University of California, San Diego School of Medicine, San Diego, CA 92103-8467 USA; 7 Department of Pathology, University of California, San Diego School of Medicine, San Diego, CA 92103-8467 USA; 8 Present Address: Macao Polytechnic University, Macau, China

**Keywords:** Metabolomics, Pathogenesis

## Abstract

Classical metabolomic and new metabolic network methods were used to study the developmental features of autism spectrum disorder (ASD) in newborns (*n* = 205) and 5-year-old children (*n* = 53). Eighty percent of the metabolic impact in ASD was caused by 14 shared biochemical pathways that led to decreased anti-inflammatory and antioxidant defenses, and to increased physiologic stress molecules like lactate, glycerol, cholesterol, and ceramides. CIRCOS plots and a new metabolic network parameter, $\dot{{{\boldsymbol{V}}}}\!$_*net*_, revealed differences in both the kind and degree of network connectivity. Of 50 biochemical pathways and 450 polar and lipid metabolites examined, the developmental regulation of the purine network was most changed. Purine network hub analysis revealed a 17-fold reversal in typically developing children. This purine network reversal did not occur in ASD. These results revealed previously unknown metabolic phenotypes, identified new developmental states of the metabolic correlation network, and underscored the role of mitochondrial functional changes, purine metabolism, and purinergic signaling in autism spectrum disorder.

## Abstract (web summary)

Developmental changes from birth to 5-years in ASD were associated with a normal physiologic response to chronic excitatory, ATP-related signaling. An inhibitory reversal in this network occurred during typical development that did not occur in ASD.

## Introduction

Autism spectrum disorder (ASD) now affects 1 in 36 children born in the United States^1^. Despite clinical heterogeneity, all children with ASD share the three core features of difficulty with language, social communication, and restricted or repetitive behaviors or interests. With current therapy, only about 20% of children diagnosed with ASD in childhood gain independence as adults^2^. Advanced methods in biochemical genetics and metabolomics over the past 65 years have shown that many metabolic changes can be found in children and adults with ASD, but the specific differences change by age, sex, and severity of symptoms^3–9^. The developmental nature of metabolic changes in ASD has been attributed in part to a delayed maturational program^10–12^, which in turn, is coupled to corresponding developmental changes in brain structure and function, immunity, the microbiome, and the autonomic nervous system^4,13–15^. A root regulator of these multisystem changes in ASD has not yet been discovered.

Whole genome sequencing efforts have identified 134 genes and structural DNA variants in 5100 children and adults with ASD^16^. However, no single genetic change accounted for more than 0.5% of the children. Diverse environmental factors are also known to increase the risk of ASD. These range from gestational fever^17^ to early exposure to environmental pollutants^18^, but no single environmental risk factor is found in every child with ASD. Exhaustive DNA sequencing and epidemiologic studies in ASD over the past 10 years have reinforced the findings that genes do not work in isolation, and that polygenic and gene-environment interactions are the dominant contributors to the development of ASD^19^.

The conceptual framework of the cell danger response (CDR) was developed to explain how genes and environment are connected through metabolism^20,21^. The CDR is a universal response to genetic or environmental stress^21^ and describes how different genetic and environmental stressors alter development and increase the risk of ASD^22^. Starting with mitochondria and the cell, the CDR propagates from local to remote systems to coordinate the metabolic, inflammatory, autonomic, neuroendocrine, and other responses needed to heal and recover from any stress or injury, and to adapt to future exposures^23^. When triggered by chronic or repeated exposure to certain environmental pollutants or other early life stresses^24^, elements of the CDR that were acutely adaptive, become chronic and pathologic. When the triggering factors occur during gestation or early in childhood, ASD and several other neurodevelopmental disorders can result. In the case of ASD, four features of systemic stress are universally present. These include changes in mitochondrial function, oxidative stress, innate immune activation, and changes in the microbiome^25^. These are each established features of the CDR^20^.

The root regulator of the CDR is extracellular adenosine triphosphate (eATP). eATP release is triggered by both genetic and environmental factors^26^. eATP is released in a graded fashion in vesicles and through pannexin 1 and other membrane channels in proportion to the magnitude of stress^27,28^. ATP is metabolically expensive to make, and proper compartmentalization is essential for normal cell function. The intracellular functions of ATP (iATP) in metabolism are well known^29^. More universal than any gene, iATP is used as an energy carrier by all forms of life on Earth^30^. However, when released to the outside of the cell, the function of ATP shifts seamlessly from matter to information. Outside the cell, eATP becomes a signaling molecule that binds purinergic receptors^31^, and signals danger. eATP signaling changes metabolism and initiates a cascade of events that starts with innate immunity, and spreads from acute, short-distance responses at the site of infection or injury, to chronic, long-distance responses that affect organ systems and regulate child development^32^. eATP is one of the most powerful signaling molecules known, capable of binding to receptors found on every cell in the body and regulating their function. Acute injection of eATP profoundly regulates mitochondria and over 200 molecules in the metabolome^33^. When given during pregnancy in a mouse model, a single injection of eATP causes life-long neurodevelopmental changes and ASD-like behaviors in the offspring^34^. Specific aspects of the CDR are triggered by specific threats like a viral infection, toxic metals, drugs, or environmental pollutants. The generalized functions of the CDR are triggered directly by eATP and its metabolites.

Differences in purine metabolism and ATP-related purinergic signaling have now been reported in every experimental model and every human study of ASD in which purines have been interrogated^5,8,34–37^. Unbiased multiomics analysis has independently underscored this discovery^38^. eATP fundamentally regulates mast cells^39^, microglia^40^, neuronal sensitization^41^, vagal function^42^, and synaptic plasticity^43^. Each of these is a known issue in ASD^44,45^. Mitochondria make over 90% of ATP used for energy and purinergic signaling. Mitochondria also serve as the information processing, early warning, and early response system that allows cells to adapt to changing environmental conditions^46,47^. The mitochondrial proteome contains the enzymes for up to 789 metabolic reactions^48^. These enzymes drive the functional changes in cells needed for growth, differentiation, healing, adaptation to stress, child development, and healthy aging. Over half of these enzymes are regulated by ATP and related nucleotides^48^, and are thus responsive to changes in the CDR that regulate ATP production and release. Chronic changes in mitochondrial functions are an established feature of ASD that are capable of remodeling the metabolic network^8^, regulating gene expression^49,50^, and shifting the trajectory of neurodevelopment^51^. In the following study, we tested the hypothesis that developmental patterns in the metabolic network of infants at risk and children with ASD can provide mechanistic insight into the developmental neurobiology of ASD.

The aims of this study were: 1) to identify the developmental patterns in metabolism that distinguish children with ASD, and 2) to identify the changes in metabolic networks that can be used as early biomarkers of risk and to gain new insights into the pathophysiologic mechanisms of ASD between birth and 5-years of age.

## Results

### Participant characteristics

#### Cohort 1—The newborn study

**Fig. 1:**
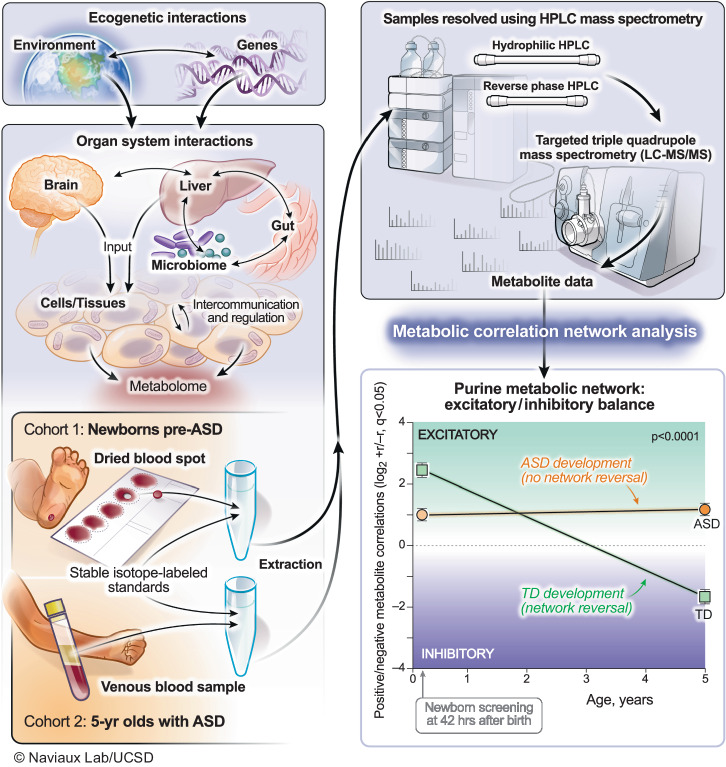
Study workflow. Metabolomics was used to interrogate real-time gene-environment interactions. The purine metabolic network was the most changed of 50 biochemical pathways analyzed in autism spectrum disorder. Developmental reversal of the excitatory (+r) to inhibitory (−r) purine network was a feature of neurotypical development. This reversal did not occur in children with ASD. ASD autism spectrum disorder, TD typically developing.

Figure 1 illustrates the biological systems interrogated and the analytical workflow of this study. Participant characteristics are shown in Supplementary Table 1. There were no differences between the pre-ASD and typically developing (TD) groups by birth weight, or the frequency of births by in vitro fertilization, gestational diabetes, or the occurrence of a gestational fever. There was also no difference in the number of medications prescribed during pregnancy, C-section, infant age at sample collection, child immunization history, maternal or paternal age, or ethnicity. We found that 48% of the children in the pre-ASD group had at least one episode of developmental regression. The frequency of regression in the TD group was 2% (*p* < 0.0001). The mean age of the first spoken word was 14.7 months in the ASD group and 11.4 months in the TD group (*p* < 0.0001). The mean age of diagnosis of ASD was 3.3 ± 1.1 years (Supplementary Table 1).

#### Cohort 2—The 5-year-old child study

Participant characteristics are shown in Supplementary Table 2. Fifty-three (53) children were enrolled in this study. All children had been diagnosed with ASD by a healthcare professional or were typically developing children enrolled as controls. This cohort consisted of children recruited prior to planned enrollment in kindergarten and before routine pre-kindergarten immunizations. The mean age was 5.3 ± 0.8 years. There were 23 males and 8 females with ASD, and 16 TD control males and 6 TD females (Supplementary Table 2).

### Metabolomics overview

#### Developmental progression of ASD-associated metabolic changes

**Fig. 2:**
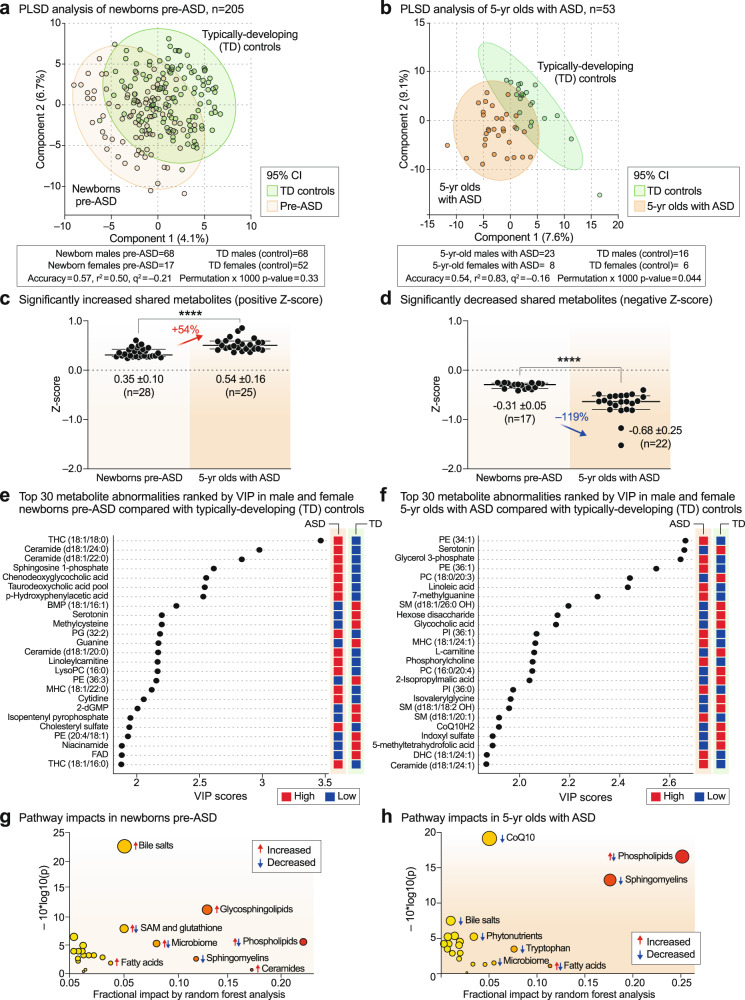
Metabolic changes in ASD. **a**, **b** Multivariate partial least squares discriminant analysis (PLS-DA). Cross-validation accuracy, *r*^2^, *q*^2^, and permutation *p* values are reported for a multivariate model with three components. **c**, **d** The magnitude of metabolic differences between ASD and TD increased with age. ****p < 0.0001. **e**, **f** Metabolite changes and pre-ASD newborns and 5-year-olds with ASD ranked by variable importance in projection (VIP) scores**. g**, **h** Bubble impact plot summary of biochemical pathway changes ranked by random forest analysis and mean decrease in accuracy (MDA). *n* = 205 newborns (68 males and 17 females in the pre-ASD group. 68 males and 52 females in the TD group), *n* = 53 5-year-olds (23 males and 8 females with ASD. 16 males and 6 females in the TD group).

Multivariate partial least squares discriminant analysis (PLS-DA) showed that newborns who later developed ASD could not be completely separated from TD newborns using classical metabolomics (Fig. 2a). The metabolic differences between 5-year-olds with ASD and TD controls allowed for greater separation, but still showed significant overlap (Fig. 2b). The top 60 most discriminating metabolites were aggregated into pathways by variable importance in projection (VIP) scores ≥ 1.5 for comparison of the metabolic impacts in newborn and 5-year-old groups. Fourteen of 24 dysregulated metabolic pathways (58%) were shared by pre-ASD newborns and 5-year-old children with ASD (Supplementary Fig. 1a, b, shaded pathways). Raw metabolomics data for newborns and 5-year-olds are reported in Supplementary Data 1 and 2, respectively. Detailed statistical analysis is reported in Supplementary Data 3 and 4. The pathways shared between the two cohorts accounted for 79% of the metabolic impact in newborns and 80% of the impact in 5-year-olds. Altered lipid metabolism accounted for 63% and 71% of the fractional impact in the newborn pre-ASD and 5-year-old ASD cohorts, respectively. About 25% of the impact was produced by changes in sphingolipids like sphingomyelins, ceramides, and glycosphingolipids, and another 20–26% was produced by changes in phospholipids (Supplementary Fig. 1a, b). See Supplementary Results and Discussion for additional details.

Quantitatively, the magnitude of metabolic disturbances in 5-year-olds with ASD was greater than in the pre-ASD newborns (Fig. 2c, d). The mean z-scores for metabolites that were significantly increased (+z-score and VIP ≥ 1.5) or decreased (-z-score and VIP score ≥ 1.5) were compared in the pre-ASD newborns and 5-year-olds. We found that the metabolites that were increased in newborns were increased by 54% more in the 5-year-old cohort (*p* < 0.0001; Fig. 2c, Supplementary Data 3 and 4). Molecules that were increased in ASD included several stress response metabolites like lactate, alanine, glycerol, glycerol-3-phosphate, threonine, linoleic acid, linoleylcarnitine, cholesterol, ceramides, and the mRNA capping purine 7-methylguanine. Metabolites that were decreased at birth were decreased by 119% more in 5-year-olds (*p* < 0.0001; Fig. 2d). Molecules that were decreased in ASD were anti-inflammatory, 1-carbon, and antioxidant molecules like glutathione, carnosine, carnitine, betaine, 5’-methyltetrahydrofolic acid, and CoQ10, and neurotransmitters like dopamine and serotonin.

#### Metabolite changes in pre-ASD newborns

The top 4 most dysregulated metabolites in the pre-ASD newborns were sphingolipids (Fig. 2e). Sphingosine-1-phosphate (S1P), ceramides and glycosphingolipids, like the mono and trihexosylceramides (MHCs and THCs), were increased, and their parent molecules, the SM lipids (sphingomyelins), were decreased. Two conjugated bile acids, chenodeoxyglycocholic acid and taurodeoxcycholic acid, were increased. Four of 27 purines were dysregulated in newborns who later developed ASD. The mRNA capping purine, 7-methylguanosine was increased, and guanine (Gua), dGMP, and AICAR (5-aminoimidazole-4-carboxamide ribonucleotide) were each decreased compared to TD controls (Fig. 2e, Supplementary Data 3). Serotonin, dopamine, and the levels of two B vitamins, niacin (niacinamide, vitamin B3) and FAD (from riboflavin, vitamin B2), were decreased in pre-ASD newborns (Fig. 2e and Supplementary Data 3).

#### Metabolite changes in 5-year-olds with ASD

**Fig. 3:**
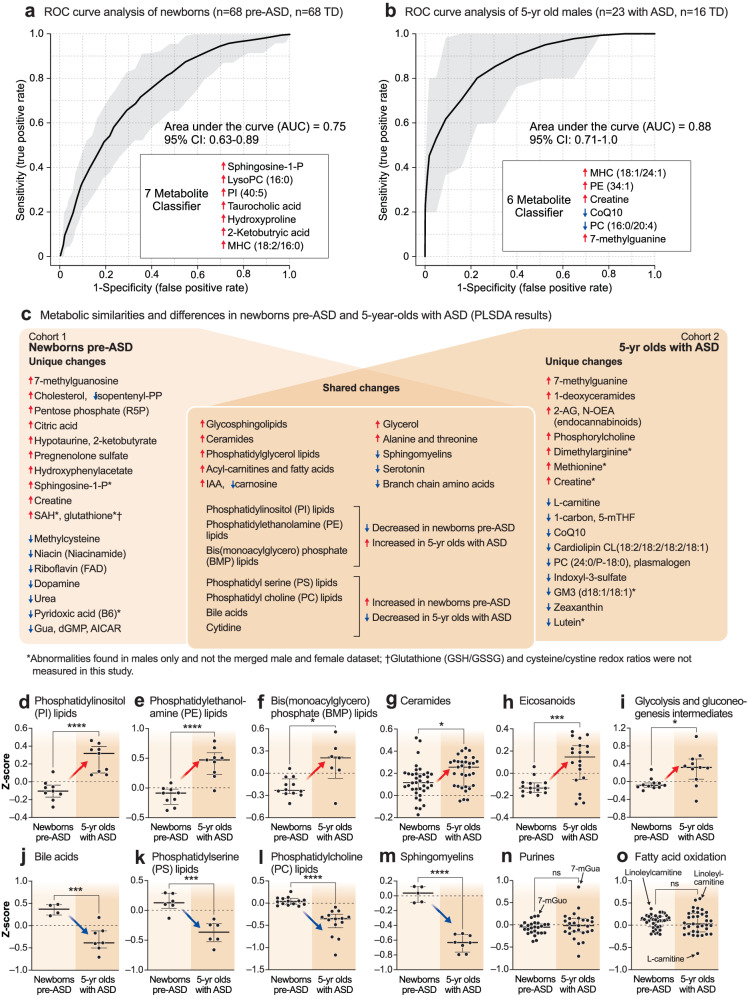
Discriminating metabolites. **a** Receiver operator characteristic (ROC) curve analysis by random forest analysis. Newborn males. *n* = 136 newborn males (n = 68 ASD and 68 TD). **b** 5-year-old males. *n* = 39 5-year-old males (*n* = 23 ASD and 16 TD). **c** Blood metabolome changes found in pre-ASD newborns are on the left. Changes found in 5-year-olds with ASD are on the right. Shared changes are shown at the intersection. *Changes found in males only. †Glutathione (GSH/GSSG) and Cysteine/Cystine redox ratios were not measured in this study. Developmental regulation of discriminating metabolites, **d** Phosphatidylinositol (PI) lipids, **e** Phosphatidyl ethanolamine (PE) lipids, **f** Bis(monoacylglycero)phosphate (BMP) lipids, **g** Ceramides, **h** Eicosanoids, **i** Glycolysis, **j** Bile acids. **k** Phosphatidylserine (PS) lipids, **l** Phosphatidylcholine (PC) lipids, **m** Sphingomyelins, **n** Purines, **o** Fatty acid oxidation and synthesis intermediates. Error bars indicate the z-score medians and interquartile ranges. Red arrows indicate metabolites that were low in pre-ASD newborns and increased in 5-year-olds with ASD. Blue arrows indicate metabolites that were increased in pre-ASD newborns and low in 5-year-olds with ASD. 5-mTHF 5’-methyltetrahydrofolate, 7-mGuo 7-methylguanosine, 7-mGua 7-methylguanine, 2-KB 2-ketobutyric acid, Gua guanine, dGMP deoxyguanosine monophosphate, AICAR 5-aminoimidazole-4-carboxamide ribonucleotide, PI phosphatidylinositol lipids, PS phosphatidyl serine lipids, PE phosphatidyl ethanolamine lipids, PC phosphatidyl choline lipids, BMP bis(monoacylglycero)phosphate lipids, IAA imidazole acetic acid, FAs fatty acids, R5P ribose-5-phosphate, SAM S-adenosyl methionine, SAH S-adenosylhomocysteine, 2-AG 2-arachidonylglycerol, N-OEA N-oleoylethanolamine, GM3(d18:1/18:1) is a monosialic, trihexosyl ganglioside, PG phosphatidylglyerol lipids. *p < 0.05, **p < 0.01, ***p < 0.001, ****p < 0.0001.

The metabolomes of 5-year-olds with ASD contained increased levels of several phosphatidylethanolamine (PE) and phosphatidylinositol (PI) lipids. Phosphorylcholine, the head group of phosphatidylcholine (PC) and sphingomyelin (SM) lipids, which is produced by the action of phospholipase Cs (PLCs) and sphingomyelinases (SMSs), was increased. Several cardiolipins needed for mitochondrial biogenesis and oxidative phosphorylation were decreased in ASD (Supplementary Data 4). Several PC and sphingomyelin lipids that are substrates for PLCs and SMSs, were also decreased. A decrease in 2-hydroxy sphingomyelins like SM(d18:1/26:0 OH) and SM(d18:1/18:2 OH) needed for myelin stabilization^52^ was observed (Fig. 2f, Supplementary Data 4). The mRNA capping purine 7-methylguanine was increased in the ASD group. Serotonin, and 3 vitamins and cofactors, L-carnitine, CoQ10, and 5’-methyltetrahydrofolic acid, were decreased. In contrast to increased levels in pre-ASD newborns, the bile acids glycocholic and chenodeoxyglycocholic acid, and the microbiome product indoxyl-3-sulfate were decreased (Fig. 2f, Supplementary Data 4). The most dysregulated metabolic pathways found in newborns who later developed ASD and 5-year-olds with ASD are summarized in Fig. 2g, h. Using metabolic classifiers of 6-7 biomarkers, the use of metabolomics was able to discriminate pre-ASD from TD newborns with an accuracy of 75%, and with an accuracy of 88% in 5-year-olds (Fig. 3a, b). A summary of the metabolic changes that were unique and shared between the two cohorts is summarized in the Venn diagram in Fig. 3c. Additional metabolomic results from classical methods of area under the curve (AUC) and z-score analysis are reported in the Supplementary Results.

### Developmentally regulated metabolic changes

The z-scores of several important classes of metabolites that were significantly changed by VIP scores ≥1.5, were found to be changed in opposite directions in the newborn pre-ASD and 5-year-old ASD groups. The top 6 classes of metabolites that were changed in newborns were either increased or underwent a developmental reversal in 5-year-olds. These were PI, PE, and BMP (bis(monoacylglycero)phosphate) lipids, ceramides, eicosanoids, and products of glycolysis like lactate and glycerol-3-phosphate (Fig. 3d–i). The top 4 classes of molecules that were decreased with age in ASD compared to TD controls were bile acids, PS and PC lipids, and sphingomyelins (Fig. 3j–m). Notably, purines and fatty acid oxidation intermediates were not dysregulated as a group. However, individual purines like the mRNA capping purines 7-methylguanosine (7-mGuo) and 7-methylguanine (7-mGua, Fig. 3n), and the polyunsaturated long-chain acyl-carnitine linoleylcarnitine, were elevated in both groups, and L-carnitine was decreased in the 5-year-olds with ASD (Fig. 3o).

### Metabolic network studies

We next explored how changes in the network of pair-wise correlations between metabolites might predict the chance of future ASD in newborns or provide new insights into the biology of ASD in 5-year-old children.

### CIRCOS analysis

#### Newborn correlation networks

**Fig. 4:**
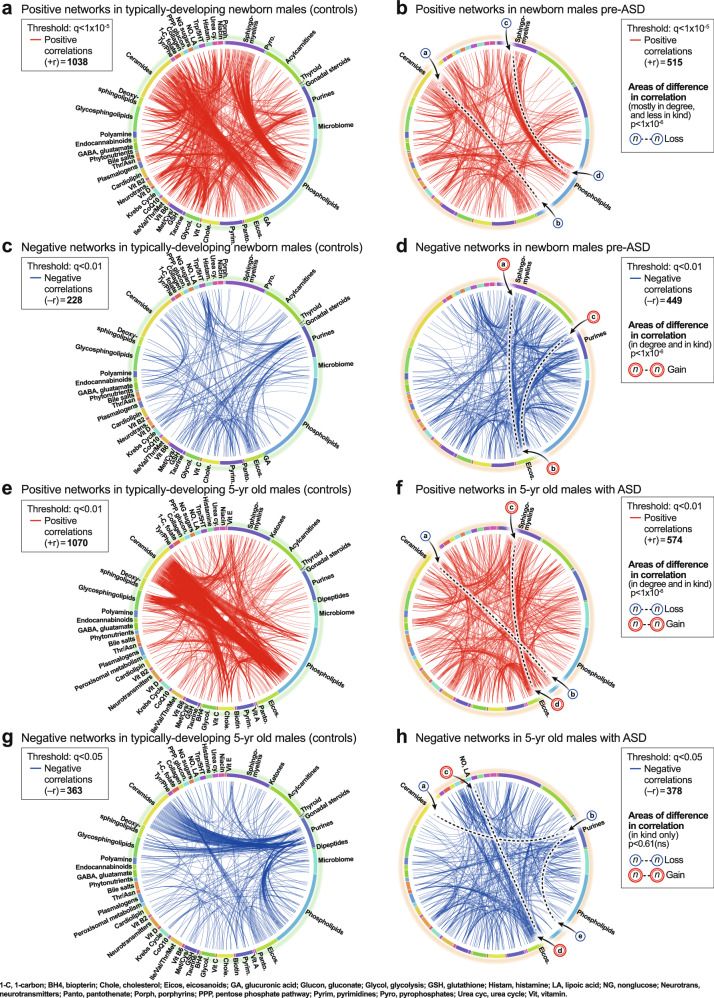
CIRCOS diagrams of the metabolic networks in typically developing controls and ASD. **a**, **b** Positive correlation networks (+r), TD and pre-ASD in newborn males. *q* < 1 × 10^-5^. **c**, **d** Negative correlation networks (-r), TD and ASD newborn males. *q* < 0.01. **e**, **f** Positive correlation networks (+r), TD, and ASD in 5-year-old males. *q* < 0.01. **g**, **h** Negative correlation networks (−r), TD and ASD in 5-year-old males. *q* < 0.05. *n* = 68 pre-ASD newborn males and 68 newborn TD male controls. *n* = 23 5-year-old ASD males and 16 TD male controls. See Results for description of the specific metabolite-pair correlations that were gained or lost.

CIRCOS plots were constructed to visualize the global metabolic networks of cases and controls. Positive (+r) and negative (−*r*) correlations were displayed separately. The global metabolic network in pre-ASD newborn males had 102% fewer positive correlations than typically developing newborns (515 vs 1038; *p* < 1 × 10^−6^; Fig. 4a, b). The sequence of metabolites around the rim of the CIRCOS plots is shown in Supplementary Data 5 and 6. Major positive correlation trunks connected ceramides and phosphatidylinositol (PI) phospholipids (segment ${ab}^{\atop\longleftrightarrow}$), and sphingomyelins and phosphatidylcholine (PC) phospholipids (segment $\overline{{cd}}$). In contrast to the loss of positive correlations in the pre-ASD network, there was a 97% gain of negative correlations in the pre-ASD group (449 vs 228; *p* < 1 × 10^−6^; Fig. 4c, d, Supplementary Data 7 and 8). Prominent among the increased negative correlations was the appearance of two new major trunks from sphingomyelins to eicosanoids (segment $\overline{{ab}}$), and from purines to the eicosanoids (segment $\overline{{bc}}$).

#### 5-year-old correlation networks

The ASD network in 5-year-olds had 184% fewer positive correlations than the TD network (574 vs 1070; 1 × 10^−6^; Fig. 4e, f). The sequence of metabolites around the rim of the CIRCOS plots is shown in Supplementary Data 6. Normal positive connections between ceramides and phosphatidylinositol (PI) phospholipids were lost in the ASD group (segment $\overline{{ab}}$). New positive connections between sphingomyelins and eicosanoids (segment $\overline{{cd}}$) occurred (Fig. 4f). There was no significant difference in the number of negative edges in the ASD and TD networks (378 vs 362 at *q* < 0.05; *p* = 0.61 (ns); Fig. 4g, h; Supplementary Data 9 and 10). However, there were several differences in the major trunks that connected metabolites. For example, normal negative correlations between ceramides and purines (segment $\overline{{ab}}$, Fig. 4g), and purines to PI lipids (segment $\overline{{be}}$) were lost in the ASD group (Fig. 4h). New negative correlations between lipoic acid in the nitric oxide/lipoic acid/ROS pathway and several eicosanoids were gained (segment $\overline{{cd}}$, Fig. 4h).

### Complementarity of network and classical metabolomic methods

We next calculated statistical parameters for all possible pair-wise correlations between metabolites in the metabolome and ranked each metabolite by its ability to discriminate between the ASD and TD networks (Supplementary Data 7–10). The network rank of a metabolite was poorly correlated with its rank determined by classical tools in metabolomics like multivariate VIP score, Random Forest mean decrease in accuracy (MDA), Welch’s adjusted *p* values, or Mann-Whitney *U* test. Classical metabolomics methods explained less than 3% of the variance in the network rank (*r*^2^ ≤ 0.03; Supplementary Fig. 2a–d). In contrast, the ranking of metabolites by classical AUC-based metabolomic methods correlated with each other (Supplementary Fig. 2e–h). Since 97% of the information obtained by network analysis could not be predicted by standard univariate or multivariate analysis of the metabolomics data, these results showed that network-based and concentration-based analyses were independent and complementary.

#### Metabolic network hub-and-spoke analysis

Based on network changes that were most significant in the 5-year-olds with established ASD, we selected the purine, ceramide, PI lipid, and eicosanoid pathways for further analysis in both cohorts.

##### The purine hub

**Fig. 5:**
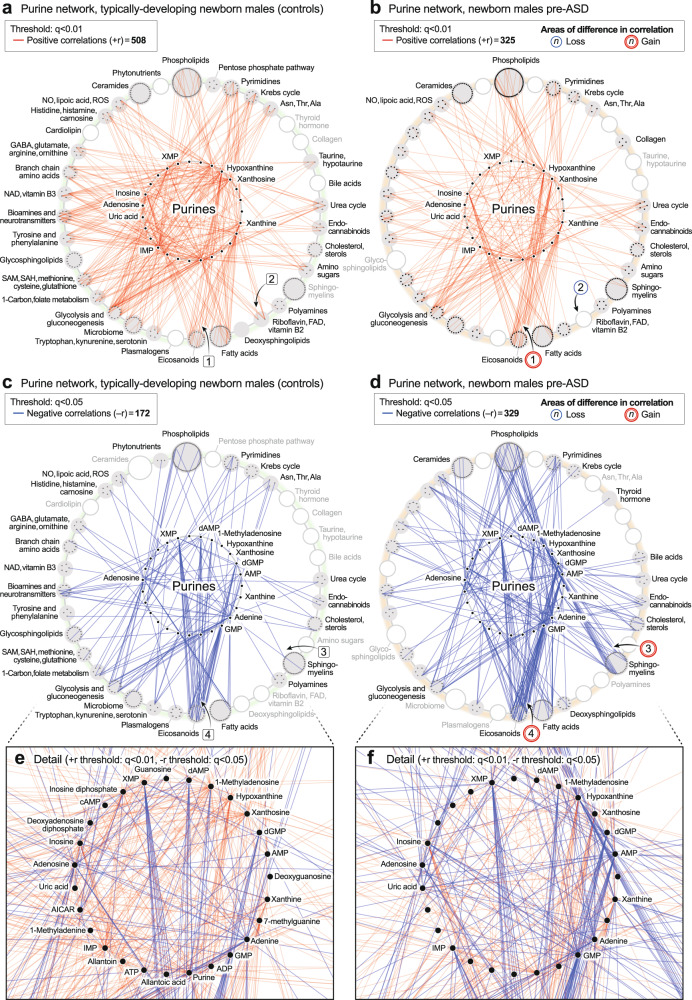
Developmental regulation of the purine metabolic network in newborn males. **a**, **b** Positively correlated (+r, red) network. **c**, **d** Negatively correlated (−r, blue) network. **e**, **f** Magnified purine hubs in newborn males. *n* = 68 pre-ASD newborn males and 68 newborn TD male controls. TD vs pre-ASD +r/−r edge ratio *p* < 0.0001; total purine network correlations *p* < 0.0003.

Unbiased network correlation analysis showed that the purine hub was the most dysregulated of all 50 metabolic pathways interrogated in the 5-year-olds with established ASD (*p* < 1.0 × 10^−22^; Supplementary Data 11 and 13). The purine network was also highly dysregulated in the pre-ASD newborns (*p* < 6.3 × 10^−22^; Supplementary Data 12 and 14). The purine hub in newborns consisted of 26 measured nucleobases, nucleosides, nucleotides, and associated metabolites. Comparing the purine hubs in TD and pre-ASD newborns, the first impression obtained from looking at the networks from a distance is that the purine hub in the TD group is relatively polymorphic, undifferentiated, and diffusely connected (Fig. 5a, e). The negative edges (−r) were few and sparsely connected (Fig. 5 c). The purine hub in the pre-ASD group on the other hand, had more structured features and appeared prematurely differentiated. New positive edges (+r) were found between xanthosine and the eicosanoids in the pre-ASD group, labeled [1] (Fig. 5b). Several purines lost positive correlations with the riboflavin/FAD pathway associated with mitochondrial fatty acid oxidation [2] (Fig. 5b). New negative correlations connected purines with several other important metabolic pathways like the sphingomyelins [3] and the eicosanoids [4] (Fig. 5d). The negative correlations between XMP and GMP and the eicosanoids were found in both the TD and pre-ASD groups (Fig. 5c, d). However, new negative correlations between AMP and the eicosanoids were found in the pre-ASD group [4] (Fig. 5d). Overall, the newborn pre-ASD purine network had fewer positive and more negative correlations than the TD network (*p* < 0.0001; Fig. 5a–d). The rise and fall of purines in pre-ASD newborn males was correlated with changes in over 400 metabolites from 31 different metabolic pathways (Fig. 5a–f).

**Fig. 6:**
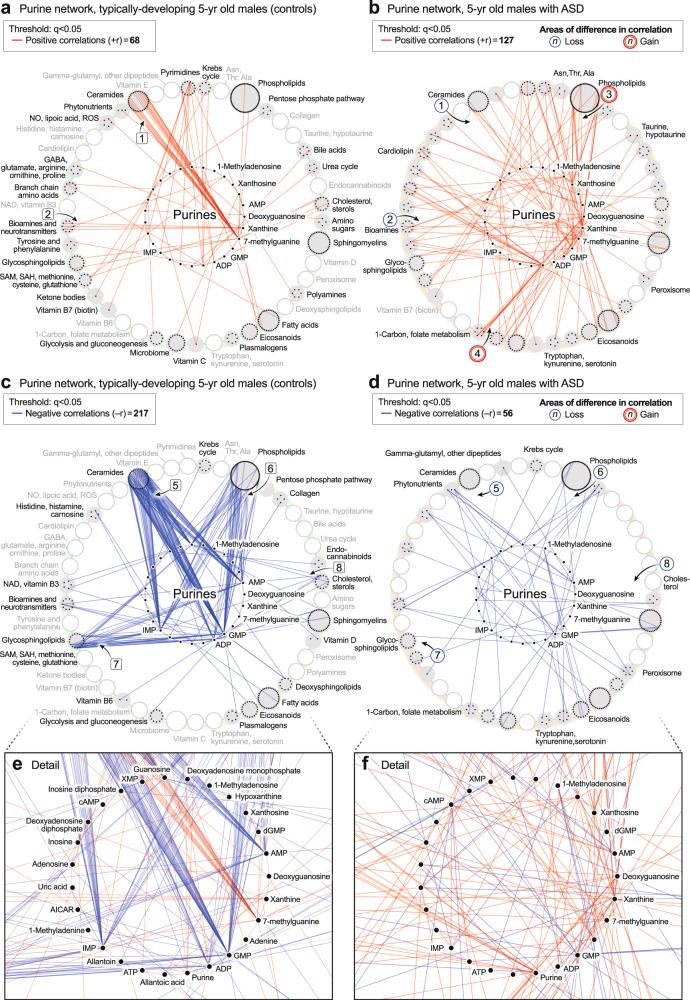
Developmental regulation of the purine metabolic network in 5-year-old males. **a**, **b** Positively correlated (+r, red) network. **c**, **d**. Negatively correlated (−r, blue) network. **e**, **f** Magnified purine hubs in 5-year-old males. TD vs ASD +r/−r edge ratio *p* < 0.0001; total purine network correlations *p* < 0.0001. *n* = 23 5-year-old ASD males and 16 TD male controls.

The purine hub in 5-year-olds consisted of 27 measured purines and associated metabolites. Comparing the purine hubs in TD and ASD groups, the first impression obtained from looking at the networks from a distance is that the purine hub in the TD group is highly structured and well-differentiated by 5 years of age (Fig. 6a, c, e). In contrast, the purine hub in the ASD group was polymorphic, diffusely connected, and developmentally underdifferentiated (Fig. 6b, d, f). The early structuring of the purine network at birth (Fig. 5b, d) led to an underdifferentiated purine network in children with ASD at age 5 (Fig. 6b, d). The ASD group lost positive correlations between 7-methylguanine and the ceramides [1] (Fig. 6b). Positive correlations were also lost between xanthine and several neurotransmitters like N-acetylaspartate and dopamine [2] (Fig. 6b). New positive correlations occurred between xanthine and xanthosine and several phospholipids [3] (Fig. 6b), and from deoxyguanosine to glycine and serine in the 1-carbon pathway [4] (Fig. 6b). The negative correlations between XMP, dGMP, and AMP, and the eicosanoids that occurred in the pre-ASD group of newborns (Fig. 5d, f), were lost by 5-years of age (Fig. 6d, f). Negative correlations between AMP, GMP, ADP, IMP, and the ceramides [5] (Fig. 6d, f), phospholipids [6], glycosphingolipids [7], and cholesterol [8] did not develop in 5-year-olds with ASD (Fig. 6c–f). The rise and fall of purines was correlated with changes in over 150 metabolites from 33 different metabolic pathways. Overall, the purine nucleobase xanthine gained the most positive correlations (+r, Fig. 6b), and GMP lost the most negative correlations (−r, Fig. 6d) of all the purines in the 5-year-old ASD metabolic network (Supplementary Data 11). New xanthine stimulatory (+r) correlations in ASD included biomarkers of mitochondrial dysfunction like myristoylcarnitine and lactic acid, and several phosphatidylserine (PS) lipids like PS(18:0/20:4) that are markers of apoptosis (Supplementary Data 9).

##### Purine network developmental arrest and failed reversal in ASD

**Fig. 7:**
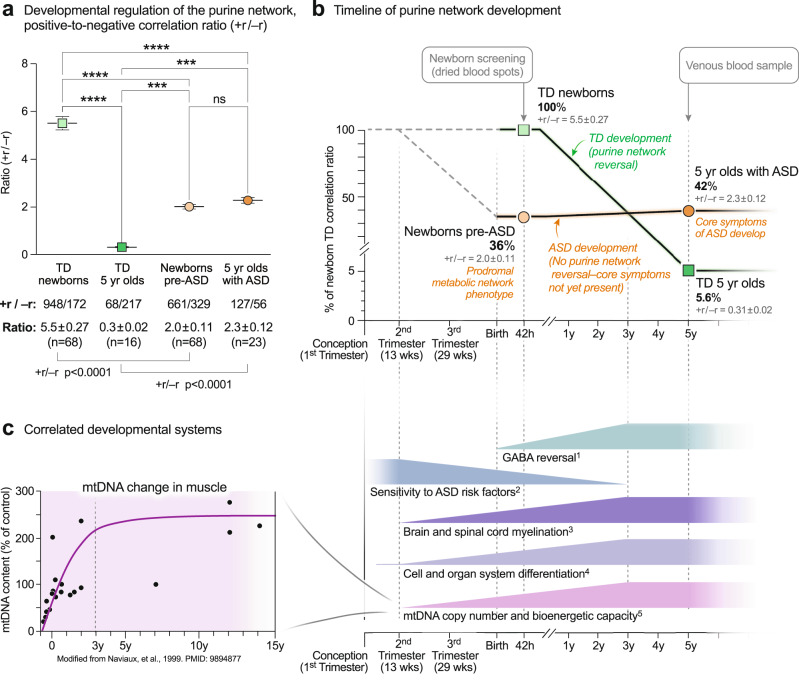
Developmental regulation of the purine network. **a** 2-way ANOVA of the +r/−r ratio. +r and −r correlations at *q* < 0.05. *p < 0.05, **p < 0.01, ***p < 0.001, ****p < 0.0001. **b** Timeline of purine metabolic network reversal. +r/−r ratios were expressed as the percent of the TD ratio in newborn males. *n* = 68 pre-ASD newborn males and 68 newborn TD male controls. *n* = 23 5-year-old ASD males and 16 TD male controls. Means are indicated in the figure. SEMs are reported numerically in gray text and were smaller than the outline of the points. **c** Correlated developmental systems. ^1^GABA (γ-aminobutyric acid) network reversal^60^. ^2^Sensitivity to ASD risk factors^18,59^. ^3^Brain and spinal cord myelination^55–57^. ^4^Cell and organ system differentiation^54^. ^5^Mitochondrial DNA (mtDNA) copy number and cell bioenergetic capacity^53^.

The ratio of positive to negative correlations—the +r/-r ratio—of purines in TD children was higher in newborns and underwent a reversal during normal development so that by 5-years of age, the +r/−r ratio was low (+r/−r = 5.5 ± 0.27 in TD newborns to 0.31 ± 0.02 in TD 5-year-olds; *p* < 0.0001; Fig. 7a, b). The magnitude of this change was 17.7-fold (5.5/0.31 = 17.7 ± 1.1). The pattern in the pre-ASD newborns was much different. The +r/−r ratio in pre-ASD newborns was just 36% of the TD group (+r/−r = 2.0 ± 0.11 in pre-ASD newborns vs 5.5 ± 0.27 in the TD newborns; *p* < 0.0001; Fig. 7a, b). In addition, the normal developmental reversal of the +r/−r ratio in the purine network did not occur in 5-year-olds with ASD. Instead, the +r/−r ratio of purine correlations in the metabolome remained statistically unchanged between birth and 5-year-old children with ASD (+r/−r = 2.0 ± 0.11 in the pre-ASD newborns vs 2.3 ± 0.12 in 5-year-olds with ASD; *p* = ns; Fig. 7a, b). To place this in physiologic context, the relationship of five correlated developmental systems is shown in Fig. 7c. Mitochondrial DNA (mtDNA) copy number increases rapidly from the 2nd trimester, continues postnatally, and nears a plateau at about age 3 years. The increase in mtDNA is associated with increased expression of the highly hydrophobic core subunits of the mitochondrial respiratory chain. These mtDNA-encoded subunits are used to nucleate assembly of the multi-subunit complexes and supercomplexes of the electron transport chain. Increased oxidative phosphorylation and increased bioenergetic capacity—the capacity for ATP synthesis—accompany the increase in mtDNA. Increasing metabolic and bioenergetic capacity permits increasing cellular metabolic specialization and differentiation^53,54^, and myelination^55–57^ (Fig. 7c). Epidemiologic studies have shown that the sensitivity to ASD risk factors like maternal infection, gestational maternal autoantibodies, pesticides, perfluoroalkyl substances (PFAS), phthalates, brominated diphenyl ether (BDE) flame retardants, and mixtures of endocrine disruptors and other potentially hazardous prenatal and postnatal environmental exposures, extends from near the beginning of the 2nd trimester, to 3 years of age^18,58,59^. The sensitivity to environmental factors and the risk of ASD decline together as the GABA signaling network reverses from net excitatory signaling at birth, to net inhibitory signaling by 2–3 years of age^60^ (Fig. 7c).

### Lipid network dysregulation in ASD

Lipids represented 58% (270 of 465) of the metabolites measured in this study. Yet we found that 80% of the TD metabolic network in 5-year-old males (4395 of 5510 edges with *q* < 0.05) was formed from nodes created by lipids (*p* < 0.0001; Supplementary Data 11). In comparison, 60% of the ASD metabolic network (2256 of 3782 edges at a threshold of *q* < 0.05) was formed from lipids (TD vs ASD *p* < 0.0001). The fraction of the metabolic network formed by polar non-lipid metabolites like purines, amino acids, and neurotransmitters doubled from 20% in TD to 40% in ASD. After the purine hub, some of the largest changes in the ASD network were hubs formed by ceramides, phosphatidylinositol (PI) lipids, and eicosanoids. These are described below.

#### The ceramide hub

**Fig. 8:**
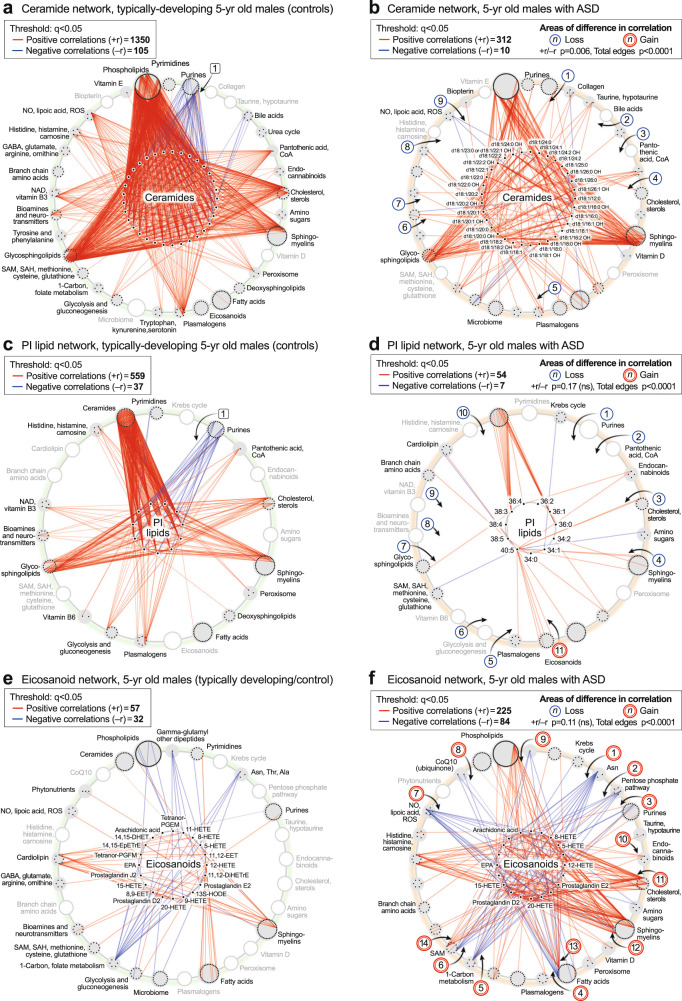
Lipid correlation networks. **a**, **b** Ceramide hub analysis. **c**, **d** Phosphatidylinositol (PI) lipid hub analysis. **e**, **f** Eicosanoid hub analysis. *n* = 23 5-year-old ASD males and 16 TD male controls. All edges with q < 0.05. EPA eicosapentaenoic acid, HETE hydroxyeicosatetraenoic acid.

Ceramide correlations were a dominant feature in typically developing 5-year-olds (Fig. 5e, g). These correlations are shown in more detail in Fig. 8a, b. The ceramide hub consisted of 33 ceramides connected by a total of 1455 edges in the TD network, but by only 322 edges in the ASD network (*p* < 0.006; Fig. 8a,b). Over 90% of the ceramide correlations (1350 of 1455 edges) in the TD network were positive (red) correlations. 100% (105 of 105) negative (blue) correlations in the TD network from the ceramide hub were to purines, labeled [1] (Fig. 8a). These negative correlations between ceramides and purines were lost in the ASD network [1] (Fig. 8b). Normal positive correlations between bile acids [2], pantothenic acid [CoA, 3], cholesterol [4], plasmalogens [5], glutamate in the neurotransmitter pathway [6], NAD+ metabolism [7], histidine, histamine, and carnosine [8], and dimethylarginine in the nitric oxide/lipoic acid pathway [9], were lost in the ceramide network of ASD (Fig. 8a, b). Strong positive correlations were maintained between ceramides, sphingomyelins, and glycosphingolipids in both TD and ASD groups (Fig. 8a, b).

#### The PI lipid hub

Phospholipids were another dominant pathway revealed by the CIRCOS analysis. The PI lipid hub consisted of 11 phosphatidylinositol lipids connected by a total of 596 edges in the TD network, but by only 61 edges in the ASD network (total edge *p* value < 0.0001; Fig. 8c, d). The PI lipid network in TD was comprised of 93% positive correlations. Most of the negative correlations (blue edges) in the TD network were to purines, labeled [1] (Fig. 8c). All the normal negative correlations between PI lipids and purines were lost in the ASD network [1] (Fig. 8d). In addition, normal positive correlations were lost in the PI network of ASD to pantothenic acid (CoA) [2], cholesterol [3] sphingomyelins [4], plasmalogens [5], glycolysis [6], glycosphingolipids [7], glutamate in the neurotransmitter pathway [8], NAD+ metabolism [9] and histidine and carnosine in the histamine pathway [10]. New positive correlations were found between the eicosapentaenoic acid (EPA; 20:5)-containing PI lipid PI(40:5) and several eicosanoids in the ASD network [11] (Fig. 8c, d).

#### The eicosanoid hub

The eicosanoid hub in ASD had nearly 4-times more positive, and nearly 3-times more negative correlations than in the TD control group (total edge *p* value < 0.0001; Fig. 8e, f). Several new negative correlations were found in the ASD network between the eicosanoids and L-asparagine [1], the pentose phosphate pathway for NADPH synthesis [2], the purine 1-methyladenosine [3], the C6-saturated medium chain acyl-carnitine hexanoyl-carnitine in the fatty acid oxidation pathway [4], 5’-methyltetrahydrofolic acid in the 1-Carbon pathway [5], cysteine-S-sulfate in the SAM/glutathione pathway [6], lipoic acid [7], and CoQ10 [8]. New positive correlations occurred between the eicosanoids and BMP(18:1/16:1) in the phospholipid pathway [9], 2-arachidonylglycerol in the endocannabinoid pathway [10], desmosterol and 7-dehydrocholesterol [11], 2-hydroxy sphingomyelins [12], docosahexaenoic acid (DHA) in the fatty acid pathway [13], and 3-methylthiopropionate from the polyamine, methionine salvage, and the SAMe pathway [14].

#### The ASD hypercorrelator hub

**Fig. 9:**
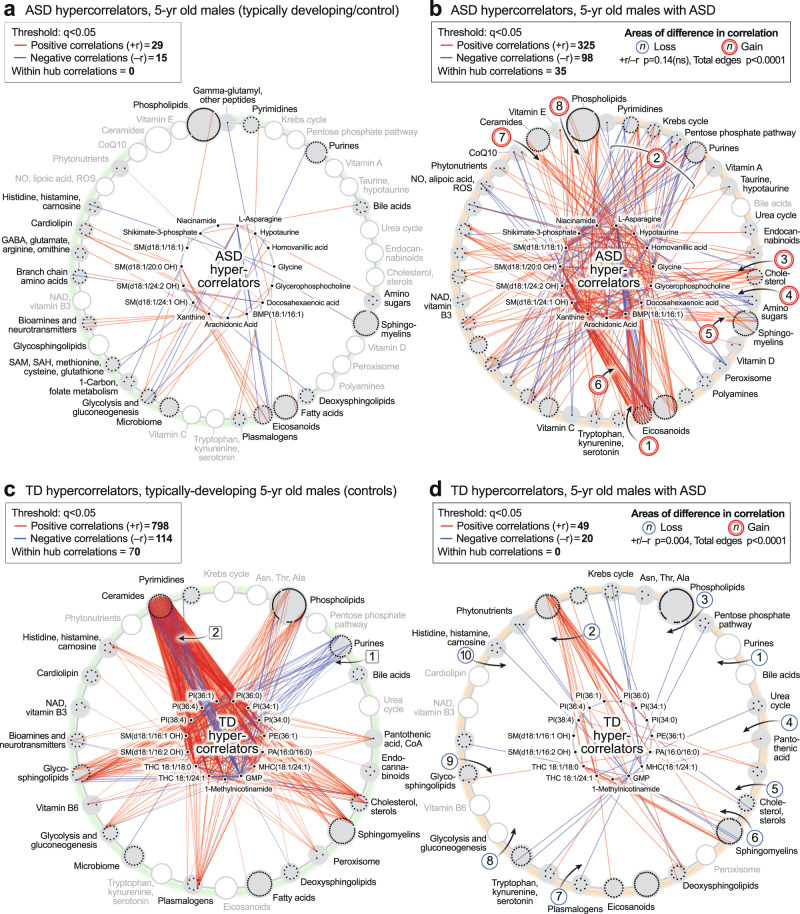
Metabolic network hypercorrelator analysis. **a**, **b** ASD hypercorrelators, 5-year-old males. **c**, **d** TD hypercorrelators, 5-year-old males. The top 15 metabolite hypercorrelators shown in each network. 467 metabolites measured. *n* = 23 ASD males and 16 TD males. All edges with *q* < 0.05.

We next studied the top 15 metabolites that were increased in the correlation network of ASD compared to TD (Fig. 9a, b; Supplementary Data 13). The ratio of positive to negative edges (the +r/−r ratio) was not different between ASD and TD (*p* = 0.14). However, the kind and degree (number) of the correlations were distinct (total edges *p* < 0.0001). A striking set of negative (blue) correlations were found between L-asparagine (Asn) and several eicosanoids like 18-, 15-, 12-, 9-, and 8-HETE, in the ASD network [1] (Fig. 8f, and the hub of 9b). Asparagine is known to be a mediator of mitochondrial signaling pathways needed for cell growth^61^ and mitochondrial ROS-mediated activation of hypoxia-inducible factor 1α (HIF1α)^62^. However, the negative correlation of asparagine with eicosanoids was a novel finding of our study. New correlations were also found between the hypercorrelators hub and pyrimidines, the mitochondrial Krebs cycle, pentose phosphate pathway, and purines [2]. Hub hypercorrelators gained new correlations with cholesterol [3], glucosamine in the amino sugar pathway [4], sphingomyelins [5], and between 2-hydroxy sphingomyelins like SM(d18:1/20:0 OH) and several eicosanoids [6], ceramides [7], and phospholipids [8]. Thirty-five (35) of 423 correlations in the ASD hypercorrelator hub (8.3%) occurred with other hypercorrelators within the hub (Fig. 9b). Similar ASD hypercorrelator analysis in the pre-ASD newborn males showed new correlations with eicosanoids, plasmalogens, androsterone-sulfate, and hypoxanthine (Supplementary Data 14).

#### The TD Hypercorrelator hub

The top 15 metabolites with the most correlations in the TD correlation network of 5-year-old males are shown in Fig. 9c, d and Supplementary Data 13. The ratio of positive to negative edges (the +r/-r ratio) was significantly different between the TD and ASD hypercorrelator networks (*p* < 0.0004). Lipids, which made up 13 of the top 15 (87%) hypercorrelated nodes in TD metabolome, lost over 90% of their correlations in the ASD metabolome (912 edges in TD vs 69 edges in ASD (92% loss; p < 0.0001). Phosphatidylinositol (PI) lipids make up 6 of the top 15 metabolites. Several negative correlations between lipids in the TD hypercorrelator network to purines [1] (Fig. 9c) were lost in the ASD network [1] (Fig. 9d). The purine GMP had multiple negative (blue) correlations with ceramides in the TD network that were lost in the ASD network [2] (Fig. 9c, d). Positive correlations that were lost in ASD occurred with phospholipids [3], pantothenic acid [4], 7-dehydrocholesterol [5], sphingomyelins [6], plasmalogens [7], glycolysis [8], and glycosphingolipids [9], and carnosine in the histidine/histamine pathway [10]. Seventy of 912 total correlations (7.7%) occurred within the hub of the TD hypercorrelator network. Similar TD hypercorrelator analysis in the pre-ASD newborn males showed new correlations with neurotransmitters like GABA and N-acetylaspartate, the Krebs cycle intermediate malate, the inhibitor of nitric oxide synthase (NOS) dimethylarginine, IMP, the gluconeogenesis metabolites phosphoenolpyruvate, dihydroxyacetone phosphate, and glycerol-3-phosphate, and the gluconeogenic amino acids proline and hydroxyproline (Supplementary Data 14).

#### Metabolic network growth ($\dot{{{\boldsymbol{V}}}}\!_{{{\boldsymbol{net}}}}$) analysis

**Fig. 10:**
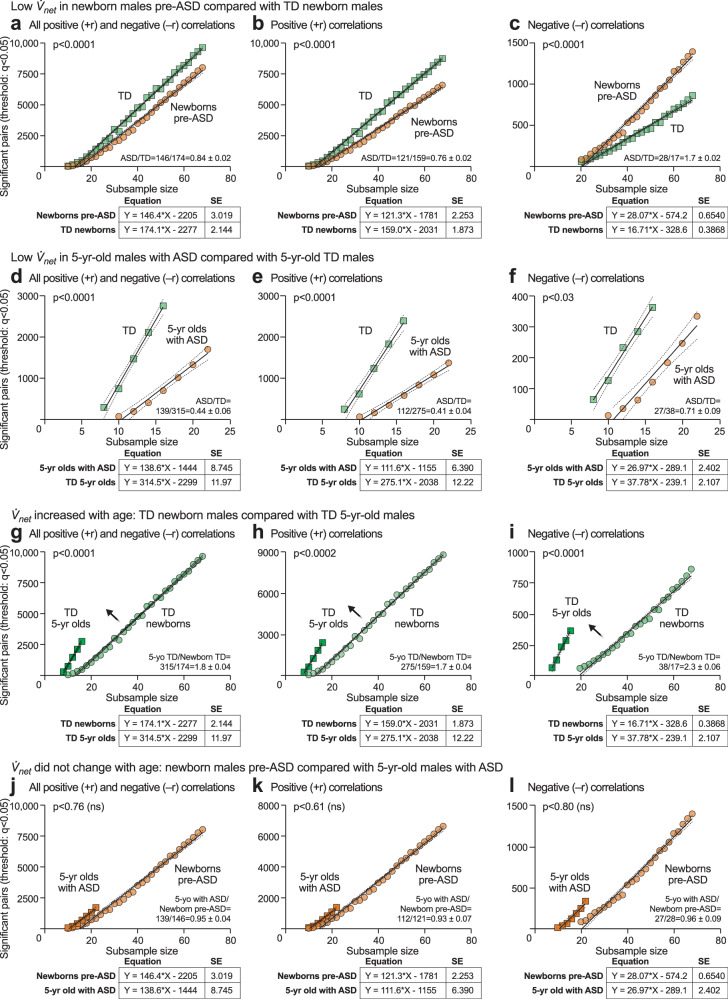
Metabolic network growth rate ( ${\dot {{{\boldsymbol{V}}}}}\!$_*net*_) analysis. Newborn males, **a–c** $\dot{{{{{{\boldsymbol{V}}}}}}}\!$_*net*_ for the positive (+r) network was decreased in newborns who later developed ASD compared to age-matched typically developing controls**. a** All +r and -r out-of-pathway correlations. **b** Positive (+r) out-of-pathway correlations. **c** $\dot{{{{{{\boldsymbol{V}}}}}}}\!$_*net*_ for the negative (−r) network was increased in pre-ASD newborns. *n* = 68 pre-ASD males and 68 TD control males. $\dot{{{{{{\boldsymbol{V}}}}}}}$_*net*_ for both the positive (+r) and negative (−r) networks was decreased in 5-year-olds with ASD compared to age-matched typically developing controls, **d** All +r and -r out-of-pathway correlations **e** Positive (+r) out-of-pathway correlations. **f** Negative (−r) out-of-pathway correlations. Green squares: TD. Tan circles: ASD. Numbers indicated are for edges with *q* < 0.05 at each indicated subsample size. *n* = 23 ASD males and 16 TD males. $\dot{{{{{{\boldsymbol{V}}}}}}}\!$_*net*_ in typically developing 5-year-old males was increased compared to typically developing newborn males, **g** All +r and -r out-of-pathway correlations **h** Positive (+r) out-of-pathway correlations**. i** Negative (-r) out-of-pathway correlations. Dark green squares: 5-year-old TD. Light green circles: newborn TD. *N* = 68 newborn males TD and 16 5-year-old males TD. $\dot{{{{{{\boldsymbol{V}}}}}}}$_*net*_ in 5-year-old males with ASD was unchanged compared to pre-ASD newborns, **j** All +r and −r out-of-pathway correlations **k** Positive (+r) out-of-pathway correlations**. l** Negative (−r) out-of-pathway correlations. Dark tan squares: *n* = 23 5-year-old males with ASD. Light tan circles: *n* = 68 pre-ASD newborn males.

Using ramped random resampling, we developed a new parameter, $\dot{{{\boldsymbol{V}}}}\!$_*net*_, to quantify the rate at which the metabolic network grows as clinical samples from the group were added (see Supplementary Fig. 3, Supplementary Table 3, and Supplementary Data 15 and 16). This method balanced statistical power by using equally sized subsamples and allowed for a comparison of the correlation network between differently sized case and control groups. Linear regression analysis showed that rates of metabolic network growth were different in ASD and TD. We found that the $\dot{{{\boldsymbol{V}}}}\!$_*net*_ in pre-ASD newborns was significantly lower than $\dot{{{\boldsymbol{V}}}}\!$_*net*_ for the TD network (+r$\,\dot{{{\boldsymbol{V}}}}\!$_*net*_ = 121 ± 2.2 in pre-ASD vs 159 ± 1.1 in TD; *p* < 0.0001; ASD/TD = 0.76 ± 0.02 at birth; Fig. 10a–c; Supplementary Data 15). The $\dot{{{\boldsymbol{V}}}}\!$_*net*_ for ASD was only 41% of the $\dot{{{\boldsymbol{V}}}}\!$_*net*_ for TD in 5-year-old children (+r $\dot{{{\boldsymbol{V}}}}\!$_*net*_ = 112 ± 6.4 in ASD vs 275 ± 12 in TD; *p* < 0.0001; ASD/TD = 0.41 ± 0.04; Fig. 10d–f; Supplementary Data 16).

#### Developmental arrest of $\dot{{{\boldsymbol{V}}}}_{{{\boldsymbol{net}}}}$ in ASD

A comparison of the ASD to TD networks revealed that the metabolic network growth rate ($\dot{{{\boldsymbol{V}}}}\!$_*net*_) in typically developing children increased by 173% between birth and 5-years of age (+r $\dot{{{\boldsymbol{V}}}}\!$_*net*_ = 159 ± 1.9 for TD newborns vs 275 ± 12 for TD 5-year-olds; *p* < 0.0001; 5-years/newborn TD = 275/159 = 1.73 ± 0.04 = 173%; Fig. 10g–i, Supplementary Table 3; Supplementary Data 15 and 16). In contrast, $\dot{{{\boldsymbol{V}}}}\!$_*net*_ in pre-ASD newborns was unchanged in 5-year-olds diagnosed with ASD, and failed to show the typical developmental increase ($\dot{{{\boldsymbol{V}}}}\!$_*net*_ = 121 ± 2.2 in pre-ASD newborns vs 112 ± 6.4 in 5-year-olds with ASD; *p* = ns; 5-years-old ASD/pre-ASD newborns = 121/112 = 1.1 ± 0.04; Fig. 10j–l, Supplementary Table 3; Supplementary Data 15 and 16).

## Discussion

Metabolism and child development are inextricably connected. Classical metabolomic analysis by laboratories around the world has shown that children with ASD have distinct metabolic profiles that vary by age, sex, and severity of symptoms^3–8^. The developmental neurobiology of ASD is driven in part by the patterned changes in metabolism that occur during child development. Using classical methods of concentration-based mass spectrometry and new methods in network metabolomic analysis, we found that the top discriminating changes in 5-year-olds with ASD occurred in phospholipid, fatty acid oxidation and acyl-carnitines, cardiolipins, ceramides, sphingomyelin, and glycosphingolipid metabolism. Fourteen pathways shared between pre-ASD newborns and 5-year-olds with ASD accounted for 80% of the metabolic impact. The metabolic phenotype of ASD was characterized by a decrease in anti-inflammatory and antioxidant molecules like glutathione, carnosine, 5’-methyltetrahydrofolic acid, and CoQ10, and a coordinated increase in stress response metabolites like lactate, glycerol, alanine, threonine, cholesterol, and ceramides. The magnitude of these metabolic changes increased with child age.

A major result of this research was that the developmental differences observed in ASD were not the result of an increase or decrease of one causal metabolite, or an isolated change in the gut-brain axis, or neuroendocrine, autonomic, cytokine, or immunologic circuits. Instead, it was the interconnectedness and developmental state of the metabolic network that underlies all these systems that was fundamentally changed. The implication of this finding is that the metabolic changes found in children with ASD were not the result of cell dysfunction or damage. Instead, the measured changes were the result of normal physiologic and neurodevelopmental responses to metabolic signals that cells received in ASD that were not being sent in typically developing children.

New methods for metabolic correlation network analysis were used to reveal previously hidden phenotypes in ASD. CIRCOS and hub-and-spoke analysis provided global, and pathway-specific views, respectively. In newborns, pre-ASD males had twice as many negative correlations and half as many positive network correlations compared to the TD group. A major trunk of positive correlations between ceramides and phosphatidylinositol (PI) lipids in the TD network was diminished in the pre-ASD network. New negative correlations between sphingomyelin and eicosanoids, and purines to eicosanoids were formed in the pre-ASD group. New positive correlations developed between the eicosanoids, cholesterol, and 2-hydroxy sphingomyelins used for myelin stabilization in 5-year-olds with ASD. Eicosanoid abnormalities have been previously reported in ASD^63^.

Dysregulated calcium homeostasis is an established feature of ASD^64,65^. In the current study, there was a loss of negative correlations between ceramides and purines, and between purines and PI lipids that regulate calcium homeostasis in 5-year-olds with ASD. Ceramides and PI lipids are functionally related. These lipids co-aggregate in microdomains of membrane lipid rafts (MLRs) and mitochondria associated membranes (MAMs) in stressed cells. Cooperativity between ceramides and PI lipids is used for activation of the phosphatidylinositol-3-dependent kinase 1 (PI3K)/protein kinase B (AKT)/mammalian target of rapamycin (mTOR) pathway that is used during conditions of cell stress^66^. IP3-activated release of intracellular stores of calcium in the ER is used for stress signaling^67^, and the activation of the AKT/mTOR pathway, play key roles in the neurobiology and immunobiology of ASD^68^. The TD network was comprised of dozens of strong inhibitory connections between purines like AMP, ADP, and ATP and the PI lipids, creating a natural dampening effect that prevents runaway cell activation by IP3-mediated calcium signaling. The ASD network lacked these natural dampeners to cell activation. The loss of inhibitory correlations between purines and PI lipids has implications for poorly regulated IP3-stimulated calcium release, persistent cell activation, and hypersensitive sensory responses in ASD. Ceramides are inhibitors of mitochondrial complex III that can trigger apoptosis and inhibit PTEN-induced kinase 1 (PINK1)-dependent mitochondrial quality control by mitophagy^69^. The loss of negative correlations between purines and ceramides in ASD can result in accumulation of dysfunctional mitochondria and excessive apoptotic cell death in response to non-injurious environmental stimuli.

Of all 50 metabolic pathways interrogated, the purine correlation network was the most changed in ASD. Purine network hub analyses revealed a 17-fold reversal from excess positive (+r) correlations in typically developing newborns, to excess negative (−r) correlations in TD 5-year-olds. This reversal failed in newborns who later developed ASD. The developmental reversal of positive (+r, stimulatory) and negative (−r, inhibitory) correlations in the purine network was similar to the reversal of the effects of GABA (γ-aminobutyric acid) signaling in normal child development^70^. The GABA-A receptor is a chloride channel^71^. GABA is an excitatory and stimulatory (depolarizing) neurotransmitter at birth and early development, then becomes inhibitory (relaxing, hyperpolarizing) during postnatal development as nicotinic cholinergic safety signaling drives mitochondrial biogenesis^72^, and facilitates the shift to the mature pattern of inhibitory GABA signaling^73^. This change is mediated in part by decreased intracellular chloride (Cl^-^) concentrations that occur with normal brain development^74^. Early excitatory GABAergic activity in striatal cholinergic-GABA-ergic interneurons is required to facilitate later developmental reversal to inhibitory GABA function. If early excitation is blocked prematurely with bumetanide to inhibit the chloride importer NKCC1, then the later reversal to inhibitory GABA signaling is incomplete^75^. Interestingly, our data showed a similar pattern during maturation of the ATP-related purine network. In contrast to the TD network, infants at risk for future ASD had a decreased ratio of excitatory to inhibitory (+r/−r) correlations in the ATP network at birth, and this ratio did not decrease during ASD development.

ATP, chloride, GABA signaling, and mitochondria are fundamentally interrelated. Increasing mitochondrial bioenergetic (ATP-producing) capacity is made possible by a phase of rapid increase in mitochondrial DNA (mtDNA) copy number that occurs in cells as they develop from the 2nd trimester to 3 years of age^53,54^. As mitochondrial biogenesis and oxidative phosphorylation increase, cellular ATP reserves increase. The increased ATP reserve creates increased capacity of the sodium-potassium ATPase (Na^+^/K^+^ ATPase) pump and permits more rapid reestablishment of ion gradients and membrane potential after depolarization. Intracellular chloride concentration is decreased as mitochondrial biogenesis continues and neurons mature. This occurs in part because of decreased expression of the bumetanide-sensitive, sodium, potassium, and chloride co-importer NKCC1 and increased expression of the chloride-extruding potassium-chloride cotransporter KCC2^76^. Combined with higher intracellular potassium produced by a more active Na^+^/K^+^ ATPase, the increase in KCC2 expression enables the developmental reduction in intracellular chloride. These data support the hypothesis that failure of the normal reversal of the purine network underlies the failed reversal of the GABA signaling network, and underlies the imbalance between excitatory-inhibitory circuits in ASD that was first described by Rubenstein and Merzenich^77^. The discovery of developmental arrest of the purine network creates a strong biological foundation for the increasing number of pre-clinical studies^34,36,78–80^ and randomized clinical trials^81,82^ that have shown safety and efficacy of low-dose suramin, a non-selective purinergic antagonist that treats the core symptoms and metabolic abnormalities in ASD. If confirmed in larger clinical trials, entirely new antipurinergic drugs that have a range of receptor subtype selectivities, and pannexin channel blockers that decrease the loss of eATP during chronic stress^27^, might create new options for treatment that have not existed before.

Metabolome correlation analysis led to the characterization of a new parameter, $\dot{{{\boldsymbol{V}}}}\!$_*net*_, to quantify the rate of metabolic network growth. We found that $\dot{{{\boldsymbol{V}}}}\!$_*net*_ in pre-ASD newborns was low compared to age-matched controls. $\dot{{{\boldsymbol{V}}}}\!$_*net*_ remained low in 5-year-olds with ASD. In contrast, typically developing newborns had a higher $\dot{{{\boldsymbol{V}}}}\!$_*net*_ than pre-ASD infants. By 5-years of age, the $\dot{{{\boldsymbol{V}}}}\!$_*net*_ increased by 173% in the TD group. Although the mathematical meaning of metabolic network growth rate $\dot{{{\boldsymbol{V}}}}\!$_*net*_ is clear—fewer connections were created in ASD as each new sample was added to the network—the deeper biological meaning in other disorders and across other states of health and disease, and the broader implications for child development in ASD, will require further study. One hypothesis that fits the facts is that metabolic network hypoconnectivity in children with ASD is produced by persistence of local cell danger signaling that blocks or inhibits receptivity to long-distance coordinating signals from the brain, vagal autonomic, neuroendocrine, endocrine, and immune systems. Receptivity to long-distance, coordinating signals from the brain is needed to produce phase synchronization of chemical changes across organs and subsystems. The loss of long-distance phase synchronization will have the effect of decreasing the total number of edges and the rate at which new correlations are added to the network in ASD.

The cell danger response model provides a conceptual framework for understanding the genetics, epidemiology, neurobiology, and complex multisystem developmental biology of ASD. Acute activation of the CDR triggers a cascade of responses that starts with mitochondria and the cell, then expands to coordinate every system needed to respond to the triggering stress. Acute activation of the CDR is universal and required to heal from any injury^23^. The root regulator of the CDR is eATP signaling. Once an infection is cleared, or other triggering stress has passed, normal mitochondrial oxygen consumption is restored, reactive oxygen species (ROS) and reactive nitrogen species are reduced, intracellular redox is restored, eATP release is decreased, and the CDR becomes self-limited. However, when exposure to a stress or stressors is severe or chronic, excessive eATP release can persist. The associated drain on energy and metabolic resources places the eATP-releasing cell in peril. Only cells that can decrease their metabolic losses during chronic stress can survive. We hypothesize that the observed changes in purinergic receptor expression in the brains of children with ASD^35^ and in animal models of ASD-like behavior^80^ sensitize the cell to purinergic signaling. This adaptation permits cells to survive chronic stress by lowering the amount of eATP release needed to manage the stress. The increased bioenergetic efficiency conserves precious intracellular resources and protects the cell from death. However, the cost of sensitized purinergic signaling is a decreased threshold for activating of the CDR. When the threshold for the CDR is set too low, cells become hypersensitive and react inappropriately to non-injurious stimuli. Repeated or chronic activation of the CDR by eATP suppresses mitochondrial oxygen consumption^33^. The resulting excess in dissolved oxygen concentration within the cell causes mitochondrial and cellular redox changes that remodel the lipid composition of cellular membranes. Cellular cholesterol is biophysically concentrated in phospholipid membranes that are chronically exposed to high concentrations of dissolved oxygen. This enables mitochondrial and cell membranes to buffer excess intracellular oxygen by absorbing it from the aqueous environment of the cytosol and sequestering it in the cholesterol-enriched lipid environment of cellular and organellar phospholipid membranes^83^. The degree of cholesterol and ceramide accumulation in cell surface membranes and in MAMs, acts as a rheostat that regulates future eATP release and the production of ROS in response to stress during development^84,85^. Cholesterol and ceramide accumulation also leads to stiffened cell membranes, impaired mitochondrial fusion-fission dynamics, oxidative stress^86^, altered synaptogenesis^87,88^, and delayed and amplified responses to environmental stimuli in ASD^89,90^.

During neurotypical development, inhibitory correlations between purines and lipids develop that prevent excessive calcium activation and prevent overexcitation in response to environmental change. These self-calming connections in metabolism failed to develop in ASD. The natural consequence of the loss of these metabolic safeguards to overexcitation is for children with ASD to seek sameness to avoid the anxiety produced by change^91^, and to be more sensitive to environmental changes across many sensory domains. These include increased sensitivity to both exteroceptive and interoceptive stimuli. From an exteroceptive perspective, children with ASD have heightened sensitivity to subtle somatosensory changes to touch, new tastes, textures, colors, and new patterns in a changing environment^92^. From an interoceptive perspective, children with ASD have heightened sensitivity to adverse childhood experiences (ACEs)^93^, environmental pollutants^94^, triggers of innate immunity^95^, and to gastrointestinal^96^, microbiome^4^, and vagal autonomic^14^ changes.

In the current study, xanthine was the purine that gained the most stimulatory (+r) correlations in 5-year-olds with ASD. Xanthine is one of the end-products of eATP metabolism^97^. Xanthine is known to trigger a cascade of events that leads to mitochondrial network fragmentation, reactive oxygen species and reactive nitrogen species (ROS and RNS), eicosanoid (e.g., leukotriene, HETE, and prostaglandin) signaling, immune activation, anxiety-associated behaviors, and consolidates long-term aversive memories that make the animal hypersensitive to future environmental changes that warn of environmental danger, cause fear, and trigger anxiety in mice, and is elevated in the blood of adults with anxiety disorders^98^. Anxiety is a common but under-recognized problem in autism^99^. Purine nucleobases like xanthine and its derivatives are metabokines and ancient CDR signaling molecules that are conserved across species and were called *Schreckstoff*—alarm substances—by the Nobel Prize winning ethologist Karl von Frisch^100^. Our results support the finding from other studies that sensory over-responsivity (SOR) and anxiety in ASD are related but separable biological phenomena^101^. Our results suggest: 1) that SOR is the result of chronic hypersensitivity of the eATP signaling network that leads to brittle calcium release in response to sensory stimuli, and 2) anxiety is the result of acute effects of the metabolic byproduct of eATP, xanthine, acting as an interoceptive mediator, producing ROS^102^, fragmenting mitochondria^98^, and acutely perturbing the metabolic network in ASD.

New methods of pre-symptomatic risk stratification for ASD during newborn screening and early well-baby checks create the opportunity to identify infants at increased risk even before the first behavioral symptoms occur. Several new methods might be combined to increase the accuracy of screening for the risk of developmental delays. For example, maternal blood metabolomics^103^, maternal chemokines and cytokines^104,105^, maternal autoantibodies^106^, newborn dried blood spot metabolomics, cytokine analysis^13^, infant hair analysis^107^, and others^108^, have each shown efficacy. And because the complex chemistry of mother’s milk for nursing infants in the first few months of life integrates a number of environmental stresses, physiologic factors, and biological needs of the child, metabolomic and exposomic analysis of milk has been shown to have about an 80% accuracy in identifying children at risk for neurodevelopmental delay, including children who later develop the ASD phenotype^109^. If these combined methods are successful, then early ASD diagnosis and intervention can be used not only to improve outcomes^110^, but also to document the decrease in the incidence of ASD made possible by multimodal infant screening and intervention programs.

This study has some limitations. In the newborn cohort, we were unable to recruit sufficient females with ASD to perform an analysis of the metabolic network in females at birth. The number of females with ASD and TD controls in the 5-year-old cohort was also small and insufficient for analysis. Cross validation of the partial least squares discriminant analysis results showed that the separation between ASD and TD groups was incomplete. The negative q^2^ values showed that the global metabolome was not the only predictor of the developmental outcome. Random forest methods were used to select the most discriminating metabolites, minimize the risk of overfitting, and to aggregate metabolites into pathways to gain biological insight. Classical z-score analysis was used for metabolite quantitation. Z-score results are quantitative but relative and based on a comparison of mass spectral areas under the curve (AUCs) in case and control groups. The absolute concentrations of metabolites in micromoles per liter in the two cohorts and two sample types—dried blood spots and plasma—were not measured. Another limitation is that in contrast to concentration-based metabolic phenotypes, which can be used to identify *individuals* who later develop ASD, metabolic network parameters calculated from a group of samples measured a single time point, identify a *group* phenotype. The group correlation phenotype illuminates the shared biology but does not yet allow the identification of individuals at risk. Longitudinal data from samples collected over time from the same subject are needed to calculate the metabolic network parameters for an individual. This was a single-sample-per-patient, non-prospective study, so time-series data were not available. This was also a non-interventional study, so data in response to treatment were not available. Future prospective studies will allow the developmental transitions in metabolism and the metabolic network to be studied in real time. Interventional studies, with accompanying metabolomic, neurophysiologic, electrophysiologic, behavioral, and pharmacologic analyses will help to confirm the practical importance of purinergic signaling, and its normalization, in ASD. The proposed mechanistic studies have been hampered by the lack of FDA-approved, antipurinergic drugs available for human use.

By studying both newborns and 5-year-olds, the developmental patterns of metabolism and metabolic network formation were highlighted in this report. Classical concentration-based metabolomics and new metabolic correlation network analyses provided complementary insights into the biology of autism spectrum disorder. The classical methods showed that metabolites and pathways associated with decreased anti-inflammatory and antioxidant defenses, and increased bioenergetic stress response molecules were markers of the pre-ASD phenotype at birth and of ASD in 5-year-old children. The new correlation network methods showed that of the 50 biochemical pathways interrogated, the purine network was the most changed. In typically developing children, the purine network underwent a 17-fold reversal between birth and 5-years of age. This reversal did not occur in ASD. In typically developing children, negative correlations between purines and phosphatidylinositol (PI) lipids developed that were hypothesized to prevent or dampen spikes in intracellular calcium release in response to commonly encountered, non-injurious environmental stimuli. In addition, negative correlations between purines and ceramides developed in typically developing children that were hypothesized to prevent excessive apoptotic cell death in response to non-injurious environmental stimuli, and to support mitochondrial quality control through PINK1-dependent mitophagy. These metabolic safeguards to overexcitation did not develop in children with ASD. The failed reversal of purine correlation network was reminiscent of, and may underlie, the failed excitatory-to-inhibitory GABAergic signaling reversal and the excitatory-to-inhibitory imbalance model of ASD by Rubenstein and Merzenich^77^.

These findings support the hypothesis that the absence of the developmental reversal of the eATP signaling and purine networks in ASD leads to dysregulated calcium homeostasis and brittle regulation of the cell danger response. If these metabolic changes drive differences in child development, then further research and development of new antipurinergic medications and devices designed to normalize the hypersensitive cell danger response may one day help to improve developmental outcomes and create new options for treatment and services for children and adults with ASD.

## Methods

### Study design

Children in the newborn cohort were enrolled when they were between the ages of 3 and 10 years and their newborn and demographic data studied retrospectively. After determining if the child had ASD or was typically developing at the time of enrollment, we received permission from the parents or guardians to go back in time to analyze their dried blood spots that were collected at birth and archived as part of the California newborn screening program^111^. Both conventional metabolomics and new methods for metabolic network analysis were used. The cohorts were analyzed first by combining data that included samples from both males and females. Next, males were analyzed separately. Fewer females with ASD were enrolled (17 females in the newborn cohort, and 8 in the 5-year-old cohort). Detailed analysis of females with ASD, separate from males, was underpowered statistically, and was not performed. All network studies were performed using data from males only. Future studies will be needed to enroll a larger number of females with ASD for sex-specific metabolomic and network analysis.

### Participant inclusion and exclusion criteria

Cohort 1 consisted of participants in a newborn screening study designed to test if a latent signature of the pre-ASD phenotype can be detected at birth. This study utilized dried blood spots collected at birth as part of the universal newborn screening program in California^111^. Secure responses to the study questionnaire were collected online using REDCap (https://projectredcap.org/about/). Signed informed consent was obtained from the parents or guardians of all participants. Inclusion criteria were a DSM-IV diagnosis of autism spectrum disorder in children between the ages of 3 and 10 years, or typically developing controls 3-10 years old. Exclusion criteria were: 1) not born in California, 2) sibling already enrolled in the study, 3) non-term pregnancy duration of either <37 weeks or >42 weeks, 4) complicated labor and delivery requiring perinatal resuscitation or hospital stay over 7 days, 5) readmission to the hospital for any reason in the first month of life, 6) any chronic disease diagnosis in a typically developing control subject. Dried blood spot samples were collected at birth from 11/23/2007 to 1/29/2018. There were 68 males with ASD and 68 TD male controls (n = 136 males), 17 females with ASD, and 52 TD female controls (n = 69 females). The male to female ratio in the ASD group was 3.9 to 1. The total number of samples analyzed in this study was n = 205.

Cohort 2 consisted of a study of 5-year-old children with ASD or TD controls. The children were originally recruited as part of the CHARGE (CHildhood Autism Risks from Genetics and Environment) Study. The CHARGE Study is an ongoing population-based case-control study that evaluates a broad range of risk factors, including environmental chemicals, in relation to neurodevelopmental outcomes. Consenting families were enrolled in CHARGE, which is associated with the Center for Children’s Environmental Health (CCEH) and the MIND (Medical Investigation of Neurodevelopmental Disorders) Institute at the University of California at Davis as described previously^112^. The CHARGE Study protocol was approved by the institutional review board of the University of California, Davis, and the State of California Committee for the Protection of Human Subjects. Families provided written informed consent before participating. Inclusion criteria included children between 24 to 60 months old upon initial enrollment, born in California, living with at least one biological parent who spoke English or Spanish, and residing in one of the selected regional center catchment areas. No exclusions were made on the basis of genetics. An autism diagnosis was identified from records provided by the California Department of Developmental Services (DDS), which is an agency responsible for providing services to individuals with developmental disabilities via a network of regional centers throughout California. TD controls were randomly selected from the general population using birth records obtained from the California Department of Public Health Vital Statistics^112^. Children from the CHARGE study were enrolled prior to receiving their planned pre-kindergarten immunizations as part of a study of the immunologic features of ASD and TD controls. Samples were collected 8/25/2010 to 1/9/2013. In this study, the results of the baseline blood samples were analyzed. There were 23 males with ASD and 16 typically developing male controls (*n* = 39 males). There were eight females with ASD and six typically developing female controls (*n* = 14 females). The male to female ratio in this cohort was 2.9 to 1. The total number of samples analyzed in this study was *n* = 53. All ethical regulations relevant to human research participants were followed.

### Metabolomics

Dried blood spots were obtained from the California State Universal Newborn Screening and biobank program^111^ and analyzed as previously described^113^ with minor modifications. Briefly, Dried blood spots were received on Whatman 903 protein saver cards. A single 3.0 mm (~1/8th in.) punch was made using a Harris Uni-core device and transferred to a 1.7 ml Eppendorf tube. 36 µl of water and 4 µl of internal standard mix were added, mixed, and the samples stored overnight at 4 °C. Then 160 µl chilled methanol/acetonitrile (50:50) was added to bring the total volume to 200 µl and vortexed. Samples were incubated on ice for 15 min, then centrifuged at 16,000 *g* for 10 min. at 4 °C. The supernatant was transferred to a fresh vial and stored at −80 °C for future analysis. For the 5-year-old cohort, venous blood was collected in acid citrate dextrose solution A vacutainer tubes (BD# 364606), mixed by gentle inversion, then centrifuged at 2100 rpm (810 × *g*) for 10 min at room temperature. Citrate-anticoagulated plasma was then removed from the top layer, aliquoted into cryotubes and stored at −80 °C until analysis. Targeted, broad-spectrum, metabolomic analysis of 690 metabolites was performed by high performance liquid chromatography using a Shimadzu LC-20AD UHPLC system coupled by electrospray ionization to a SCIEX Qtrap 5500 triple quadrupole mass spectrometer (LC-ESI-MS/MS) as described^114^ with minor modifications to accommodate an expanded list of targeted chemicals. MS/MS detection was performed using scheduled multiple reaction monitoring (MRM) of precursor and product ions. Compound-specific source and fragmentation parameters for 2–6 MRMs for each targeted compound, along with MS/MS spectral data, and retention times by hydrophilic interaction liquid chromatography (HILIC) and reverse phase (RP) chromatography, were optimized using purified standards^114^. A total of 431 (dried blood spot study) or 467 (plasma study) of the 687 targeted metabolites were measurable in both males and females. These metabolites, their associated retention times, and MRM data are reported in Supplementary Data 19. 100% of samples provided AUC data on these chemicals. There were no missing values, and no data were imputed. This targeted metabolomics platform interrogated 46 (newborn screening) and 53 (5-year-olds) different biochemical pathways and permitted analysis of many of the metabolites known to be core features of the CDR and integrated stress response (ISR)^21,115,116^.

### Metabolic network and hub-and-spoke analysis

Metabolic network results were for the males only. Insufficient females were enrolled for correlation analysis. Metabolite AUCs were log-transformed and converted to z-scores. Pair-wise Pearson correlations between the z-scores were then calculated. The sign (+ or −) and strength (r value) of the correlation coefficient, q-values, false discovery rates (FDR), and *p* values were calculated for all the possible pair-wise metabolite correlations. To minimize overfitting, only correlations with *q* values < 0.05 were tallied. Correlations among the metabolites belonging to the same pathway were designated as in-pathway, or “in” for short. Correlations between metabolites belonging the different pathway were designated out-of-pathway, or “out” for short. About 5% of the correlations were in-pathway, and the other 95% were out-of-pathway. Metabolites were the nodes, and the pair-wise correlation was displayed as an edge in the network. Each metabolite was assigned to one of 50 biochemical pathways. The number of positive correlations, negative correlations, in- and out-of-pathway correlations, mean and median correlation strengths, and corresponding statistical parameters were calculated. CIRCOS^117^ was used to visualize the global metabolic connectome. MetScape 3.0, a plug-in for Cytoscape 3.8.2 was used to visualize the metabolic hub and metabolite connections for the most dysregulated pathways^118^.

### Network growth ($\dot{{{\boldsymbol{V}}}}\!_{{{\boldsymbol{net}}}}$) analysis

A ramped random resampling method was developed to study the properties of the metabolic network. This method permitted control for changes in statistical power produced by unequal case and control sample sizes. Pearson’s *r*, *p*, FDR, and *q* values were calculated for all possible pair-wise correlations between measured metabolites using the python package scipy v1.9.1. All correlations with *q* value ≤ 0.05 were considered significant and counted. A subset size starting at four samples, ramping to *n*−1 samples, was incremented by 2 (e.g., 4, 6, 8… to 21 for 22 controls and 4, 6, 8… to 30 for 31 cases). Subsamples from cases and controls were randomly selected using the random.sample function in python. Results obtained by ramped random resampling with and without replacement were compared and reported in the Supplementary Figs. 4–6 and Supplementary Data 15–18. Fifty (50) random samples at each subsample size were taken to estimate the population statistics based on the central limit theorem. In some cohorts, fewer than 51 samples were available and the maximum subsample size was limited to *n*−1 samples as described above. Mean, median, standard deviation, standard error of the mean (SEM), and standard error coefficients of variation defined as the SEM/mean for the significant correlations were calculated at each subsample size across all iterations. The number of significant metabolite pairs (edges) was plotted on the y-axis as a function of the subsample size on the x-axis. Least mean squares linear regression was used to identify the best fit equation for the line, *y* = *mx* + *b*, and to test the differences between the slopes for cases and controls. The slope (*m*) of the metabolic network growth curve was used as a new parameter, $\dot{{{\boldsymbol{V}}}}\!$_*net*_. The computer code for executing this analysis is available on the GitHub site for this paper. See Supplementary Methods for additional details.

#### Hypercorrelator analysis

The top 15 ASD hypercorrelators were identified by ranking all 467 measured metabolites by the total difference in out-of-pathway correlations (edges) meeting a *q* value of <0.05 and calculating the difference of ASD-TD. The top 15 had the most positive (+) differences between ASD and TD. Reciprocally, the top 15 TD hypercorrelators had the most negative (−) differences between ASD and TD. This ranking is reported in Supplementary Data 13 and 14.

#### Statistics and reproducibility

Demographic data were analyzed by t-tests or non-parametric Mann-Whitney *U* tests in GraphPad Prism (https://www.graphpad.com). Categorical data and 2 × 2 tables were analyzed by Fisher’s exact test. AUC data from metabolomics were log_2_ transformed, scaled by control standard deviations, and the resulting *z* scores were analyzed by VIP scores calculated by multivariate PLS-DA in MetaboAnalyst 5.0^119^. Cross-validation was used to calculate accuracy, *r*^2^, and *q*^2^ statistics^120^. Permutation analysis (×1000) was used to calculate the p value for the model. Mean decrease in accuracy (MDA) scores were calculated by random forest analysis from 5000 trees in R^121^. FDRs were calculated by the method of Benjamini and Hochberg^122^ and Bayesian false discovery rates by Storey *q* value^123^. Metabolites with VIP ≥ 1.5 and MDA > 0 were considered significant and were used for pathway enrichment analysis^121^. Bubble impact plots were visualized in Python. Significance was quantified as the hypergeometric *p* value and plotted on the y-axis. The k-nearest neighbor algorithm^124^, a non-parametric classification method used to cluster metabolites and pathways that behave in similar ways, was used to identify the metabolite clusters. Classifiers of 4–7 metabolites were selected and tested for diagnostic accuracy using area under the receiver operator characteristic curve and random forest analysis. Five hundred bootstrappings were used to calculate the 95% confidence intervals of the ROC curve. The 2 × 2 confusion matrix generated in MetaboAnalyst was used to calculate the sensitivity, specificity, and the accuracy of the diagnostic classifier. Diagnostic accuracy was calculated using the 1000 permutations^120^. Classifiers were validated within sample using repeated double cross-validation with bootstrapping 100 times to test random subsamples of 2/3 in and 1/3 out, and by permutation analysis^125^. See Supplementary Information for additional methods.

### Reporting summary

Further information on research design is available in the Nature Portfolio Reporting Summary linked to this article.

## A special note from the authors

Although the words “normal” and “abnormal”, “regulated” and “dysregulated”, and “functional” and “dysfunctional” are used in this paper, a major result of this research was the finding that the ASD phenotype is the result of developmental changes caused by a normal physiologic response to the signals that cells are receiving in autistic children. The cells of neurotypical children do not receive the same cell danger signals, and therefore do not show the same set of mitochondrial, metabolic, immunologic, microbiome, autonomic, neuroendocrine, neurodevelopmental, and behavioral changes. We are sensitive to the negative impact that poorly chosen words can have^126–129^ and have endeavored to use neutral, specific, and descriptive language whenever possible.

### Study approvals

This study was approved by the University of California, San Diego (#140072, #171940, #190972), California state (#1162, #2018-020), and University of California, Davis (#226004-4) institutional review boards (IRBs). Informed signed consent was obtained prior to participation.

## Supplementary information

Supplementary Information

Description of Additional Supplementary Files

Supplementary Data 1-19

Reporting Summary

## Data Availability

Metabolomics data are available in Supplementary Data 1 and 2. All numerical source data for the figures are available as a single Excel file containing tabs labeled Supplementary Data 1–19.

## Code availability

Software for metabolic network growth ($\dot{{{\boldsymbol{V}}}}\!_{{net}}$) analysis is publicly available at: https://github.com/BDNav/metabotools
