# Supplementary material for: Metabolic network analysis of pre-ASD newborns and 5-year-old children with autism spectrum disorder
Source: Commun Biol. 2024 May 10;7:536. doi: 10.1038/s42003-024-06102-y (PMC11549098; doi:10.1038/s42003-024-06102-y)
Supplement: Supplementary file 1 — Supplementary Information [file 42003_2024_6102_MOESM1_ESM.pdf]

Supplementary information for:

Metabolic network analysis of pre-ASD newborns and 5-year-old children with autism spectrum disorder

Sai Sachin Lingampelly[1,2], Jane C. Naviaux[1,3], Luke S. Heuer[4], Jonathan M. Monk[1,2], Kefeng Li[1,2], Lin Wang[1,2], Lori Haapanen[4], Chelsea A. Kelland[4], Judy Van de Water[4,5], Robert K. Naviaux[1,2,6,7*]

[1]The Mitochondrial and Metabolic Disease Center, University of California, San Diego School of Medicine, San Diego, CA 92103-8467, USA
[2]Department of Medicine, University of California, San Diego School of Medicine, San Diego, CA 92103-8467, USA
[3]Department of Neuroscience, University of California, San Diego School of Medicine, San Diego, CA 92103-8467, USA
[4]The UC Davis MIND Institute, University of California, Davis, Davis CA 95616. USA
[5]Department of Rheumatology and Allergy, School of Veterinary Medicine, University of California, Davis, Davis CA 95616. USA
[6]Department of Pediatrics, University of California, San Diego School of Medicine, San Diego, CA 92103-8467, USA
[7]Department of Pathology, University of California, San Diego School of Medicine, San Diego, CA 92103-8467, USA.

This PDF file includes:

Supplementary Results and Discussion, Methods, and References

Supplementary Tables 1-3
Supplementary Figures 1-5

Supplementary Results and Discussion

Sphingolipid abnormalities

Glycosphingolipids like mono-, di-, and trihexosylceramides (MHCs, DHCs, and THCs) were increased in both newborns at risk, and in 5-year-olds with ASD (Fig. 2g-j). The majority of dihexosylceramide, DHC(18:1/24:1), species contain the disaccharide lactose and are known as a lactosylceramide (LacCer; Galβ4Glcβ1Cer). LacCer was increased in both cohorts and plays an important role in the generalized innate immune system and the response to fungal and parasitic infections [1, 2]. Glycosphingolipids form the ABO blood group antigens [3], are critical for normal brain development, invariant natural killer T (iNKT) cell function, and for regulating host-pathogen interactions [4]. Ceramides are another class of sphingolipids that play key roles in mitochondrial quality control and apoptosis pathways. Ceramides were increased in both newborns at risk and 5-year-old children with ASD. Ceramides inhibit mitochondrial complex III [5], induce the serine/threonine protein phosphatases PP1 and PP2A. PP1 and PP2A inhibit cell proliferation by dephosphorylating the AKT/Protein Kinase B protein, releasing cytochrome c, activating caspases, and sensitizing cells to several different cell death pathways in response to stress[6, 7]. Ceramides are synthesized from sphingomyelins by stress-activated induction of membrane acid and neutral sphingomyelinases [8, 9].

Supporting evidence for the activation of sphingomyelinases in both cohorts was the increased sphingosine-1-phosphate (S1P) in the newborns at risk, and the increase in phosphorylcholine in 5-year-old children with ASD (Fig. 2h, Supplementary Data 3 and 4). S1P is made from the sphingomyelins by the sequential action of sphingomyelinase, ceramidase, and sphingosine kinase. While ceramides are pro-apoptotic, S1P is a pro-survival signaling molecule that acts by binding a dedicated G-protein coupled receptors (S1PRs 1, 2, and 3) [10]. Phosphorylcholine and diacylglycerol (DAG) are the direct products of sphingomyelinase action on sphingomyelins.

Sphingomyelinases 1 and 2 (SMS1 and 2) can also produce phosphorylcholine from phosphatidylcholine (PC) lipids in a reaction that was formerly attributed to a phosphatidylcholine-specific phospholipase C (PC-PLC), and now known to be SMS1 and 2 [11]. SMS1 and 2 trigger inflammation in response to microbial infection and release DAG to activate protein kinase C (PKC) and stimulate reactive oxygen species (ROS) production. Phosphorylcholine was increased in 5-year-old children with ASD. Sphingomyelins were decreased in both newborns at risk and the 5-year-olds with ASD, consistent with a biological response to stress at both ages.

We found new positive correlations emerge in 5-year-olds with ASD between the 2-hydroxy sphingomyelins like SM(d18:1/20:0 OH), SM(d18:24:1 OH), and SM(d18:24:2 OH), and virtually all the eicosanoids (Fig. 9b, “6”). The fatty acid 2-hydroxylase (FA2H) needed for 2-hydroxylation of the fatty acid used for the N-amide linked acyl group in sphingomyelins is a peroxisomal enzyme[12]. 2-hydroxylation is critical for myelin stabilization[13]. Since the 2-hydroxy sphingomyelins were decreased in ASD, the positive (+r) correlation with pro-resolving 15-, 8-, and 5-HETEs and eicosapentaenoic acid (EPA) (Fig. 8f, “12”) is consistent with impaired resolution of inflammation after a stressful environmental change.

Mitochondria and other shared pathway abnormalities

Mitochondrial fatty acid oxidation was dysregulated in both cohorts. In newborns at risk, this was characterized by elevations in the carnitine esters of poly unsaturated and monounsaturated, long-chain fatty acids like arachidonyl-carnitine (20:4), linoleylcarnitine (18:2), and oleoylcarnitine (18:1). Linoleylcarnitine was the most increased acylcarnitine in both the newborn and 5-year-old cohort (Fig. 4l). In the 5-year-olds with ASD, this had progressed to show an increase in the corresponding free fatty acids, linoleic and oleic acid. Free carnitine levels were also decreased in the 5-year-olds with ASD (Fig. 4l, Supplementary Data 4).

Markers of mitochondrial branch chain amino acid (BCAA) metabolism were dysregulated in both cohorts. In newborns at risk, the C6 leucine metabolite hydroxyisocaproic acid was decreased (Supplementary Data 3). In 5-year-olds with ASD, the glycine ester of the C5 decarboxylation product of leucine metabolism, isovalerylglycine, was decreased (Fig. 2h).

Stress-induced polar metabolites

Two gluconeogenic amino acids were increased. In newborns at risk, alanine was increased. Alanine can be synthesized from lactic acid oxidation to pyruvate and transamination. In 5-year-olds with ASD, alanine was not elevated. Instead, the gluconeogenic amino acid threonine was increased (Supplementary Data 3). Elevations in alanine and/or threonine are associated with physiologic responses to stress in both plants and animals [14, 15]. Another stress-induced metabolite, glycerol, was increased in both cohorts, and glycerol-3-phosphate was increased in 5-year-olds with ASD (Supplementary Data 3, Fig. 2h). Glycerol and glycerol-3-phosphate (G3P) are 3-carbon byproducts of glycolysis that are diverted for lipid storage under conditions of physiologic stress [16, 17].

Serotonin

Serotonin was decreased in both newborns at risk and 5-year-olds with ASD (Fig. 2h, Supplementary Data 3 and 4). Placental serotonin is the primary source of serotonin during early brain development in the fetus [18]. Gestational exposure to endocrine disrupting chemicals like bisphenol A (BPA) decreases placental serotonin [19]. Gestational infection or other stressors cause increased release of extracellular ATP, which binds P2X7 and other purinergic receptors, triggering IL1β and IL6 production, caspase 1 and placental NLRP3 activation [20]. The placenta acts as historical record of stresses encountered during pregnancy, preparing the fetus for the environment into which they will be born [21]. Decreased placental serotonin has led to decreased

postnatal blood serotonin levels. Post-natal exposure to endocrine disrupting environmental chemicals also decreases circulating serotonin and increases the developmental risk of ASD [22].

Phospholipids

Phospholipids were dysregulated in both cohorts. This accounted for 20% to 26% of the metabolic impact in cohorts #1 and #2, respectively (Fig. 2cd). However, we found that the subclasses of phospholipids were differentially dysregulated in newborns at risk compared to 5-year-olds with ASD (Fig. 4). For example, phosphatidylinositol (PI) lipids needed for IP3-mediated calcium signaling from the endoplasmic reticulum were decreased in newborns at risk and increased in in 5-year-olds with ASD. Phosphatidylethanolamine (PE) lipids were decreased in newborns but increased in 5-year-olds with ASD. PE lipids are needed for synthesis of endocannabinoids and for synthesizing phosphatidylcholine (PC) lipids by SAM-dependent PE methyltransferase (PEMT). Bis(monoacylglycero) phosphate (BMP) lipids needed for endosomal trafficking and used for lysosome-dependent mitophagy and autophagy pathways, were also decreased in newborns at risk but increased in the 5-year-olds with ASD. One of the most decreased PC lipids in 5-year-olds with ASD was PC(16:0/20:4), which contains the polyunsaturated fatty acid arachidonate (20:4). Arachidonate is cleaved from by phospholipase A_2 (PLA_2) under conditions of cellular stress and used to make eicosanoid signaling lipids like prostaglandins, leukotrienes, prostacyclins, and thromboxanes.

Purines

Several purines were decreased in newborns but unchanged in 5-year-olds with ASD. These included dGMP, its nucleobase guanine, and the purine precursor 5-aminoimidazole-4-carboxamide ribonucleotide (AICAR) (Supplementary Data 3). In contrast, the mRNA capping purines 7-methylguanosine (7-mGuo) in newborns, and 7-methylguanine (7-mGua)) in 5-year-old children, were increased in ASD (Fig. 4k, Supplementary Data 4). This was consistent with

a shift from *cap-dependent* housekeeping mRNA translation, to the stress-induced, PINK1-mediated (PTEN-induced kinase 1-mediated), *cap-independent* mRNA translation [23].

The pyrimidine-phospholipid-inflammation connection

Cytidine is a pyrimidine that was increased in newborns at risk and decreased in 5-year-olds with ASD. Cytidine and uridine ribosides are phosphorylated to CMP and UMP, respectively, by uridine/cytidine kinase 2 (UCK2). Recent studies have showcased the relationship between pyrimidine metabolism and the subsequent phosphorylation of CMP, dCMP, and UMP and dUMP by the mitochondrial enzyme cytidine/uridine monophosphate kinase 2 (CMPK2) and the induction of this enzyme during times of inflammation, and mitochondrial DNA synthesis [24]. Of key importance is that CMP is generated from CDP-choline and CDP-ethanolamine each time a PC or PE lipid is synthesized by the Kennedy pathway. The rephosphorylation of CMP to CDP by CMPK2, and to CTP by nucleoside diphosphate kinase/nonmetastatic (NDK/NME), ensures that phospholipid synthesis is not starved for CTP nucleotides, and membrane synthesis can continue [25].

Phosphatidylserine (PS) lipids showed an opposite pattern compared to PE lipids. PS lipids were increased in newborns at risk but were decreased in 5-year-olds with ASD (Fig. 4h, Supplementary Data 3, 4). PS lipids are decarboxylated in mitochondria to synthesize PE lipids. When PS lipids are elevated compared to PE lipids, it is consistent with a relative bottleneck at the mitochondrial enzyme phosphatidylserine decarboxylase (PISD) [26], or increased conversion of PE lipids to PC lipids for extramitochondrial membrane synthesis. PE lipids are enriched in mitochondrial membranes, interact with cardiolipin, and are essential for mitochondrial oxidative functions [27]. PE lipids were increased in the 5-year-olds with ASD.

Microbiome

Several microbiome metabolites were developmentally dysregulated in this study. Bile acids used for communication between the liver and the microbiome via the farnesoid X receptor (FXR) and the G-protein bile acid receptor 1 (GPBAR1), were dysregulated. For example, chenodeoxycholic acid was increased in the newborns at risk but decreased in 5-year-olds with ASD. Phenyllactate and hydroxyphenylacetate belong to a class of chemicals called phenylketones. Both phenyllactate and hydroxyphenylacetate, which are produced by disturbances in the microbiome and induced by systemic ATP-related purinergic signaling[28], were increased in newborns at risk (Supplementary Data 3). A maturational shift in the microbiome occurred by 5-years of age when children with ASD showed no increase in phenylketones, but showed a decrease in the key immune regulator indoxyl-3-sulfate (I3S), which is an endogenous ligand of the aryl hydrocarbon receptor (AhR) and suppresses inflammation [29]. Deficient microbiome production of I3S is associated with a pro-inflammatory state. We targeted another important microbiome product that can be made from hydroxyphenylacetate, 4-ethylphenyl sulfate (4-EPS) [30], but did not find a difference in 4-EPS between children with ASD and TD controls (Supplementary Data 3 and 4).

Histamine

The histidine/histamine pathway was dysregulated in different ways in the two cohorts. The antioxidant dipeptide carnosine (β-alanyl-L-histidine) was decreased in newborns at risk but unchanged in 5-year-olds with ASD. By 5-years of age, children with ASD showed an increase in the histamine metabolite imidazoleacetic acid (IAA) Supplementary Data 3 and 4).

Metabolic pathways in newborns associated with future risk of ASD

1-carbon, methylation, and transsulfuration pathways

Transsulfuration and methylation pathways were disturbed in newborns at risk for ASD. S-adenosylhomocysteine (SAH) and glutathione were both decreased in newborn males at risk

ASD (Supplementary Data 3). 1-carbon, methylation, and transsulfuration pathways are connected. However, an overt deficiency of folic acid, B12, or cysteine was not found when males and females were merged in the newborn analysis. Instead, we found evidence of increased turnover of cysteine in the form of an increase in its catabolites 2-ketobutyrate and hypotaurine. Methylcysteine, an important antioxidant and anti-inflammatory molecule [31], was decreased in newborns at risk for ASD (Supplementary Data 3). Deficient production of methylcysteine weakens antioxidant defenses during times of infection and physiologic stress.

Isopentenyl pyrophosphate and sterol synthesis

Isopentenyl pyrophosphate (IPP) was decreased in newborns at risk for ASD (Fig. 2g, Supplementary Data 3). IPP is an intermediate of cholesterol biosynthesis, regulated by the gene isopentenyl diphosphate isomerase 1 (IDI1). IPP is an important inhibitor of osteoclasts and activator of $\gamma\delta$-T-cells [32]. Two end products of cholesterol synthesis, cholesteryl-sulfate and pregnenolone, were both increased. Pregnenolone is neuroprotective under conditions of excitatory stress [33]. It is the first committed step in steroid hormone synthesis and is synthesized in mitochondria by the NADPH-dependent cholesterol P450 side-chain cleavage enzyme (P450scc) encoded by CYP11A1. NADPH is produced by pentose phosphate pathway (PPP) and used by NADPH oxidases (NOXs) to produce ROS under conditions of infection or other physiologic stress [34]. Newborns at risk for ASD showed an increased flux of glucose through the PPP in the form of increased ribose-5-phosphate (R5P) needed for RNA and DNA synthesis (Supplementary Data 3). NADPH was not measured directly in this study.

Mitochondrial stress response molecules

Citric acid was increased in newborns at risk for ASD (Supplementary Data 3). Citric acid, oxidized in mitochondria through Krebs cycle, utilizes NAD^+ from vitamin B3 (niacin) and FAD from vitamin B2 (riboflavin) as electron acceptors to generate NADH and $FADH_2$ and drive ATP

synthesis by oxidative phosphorylation. When NAD^+ or FAD are decreased, or the ratio of $NADH/NAD^+$ is increased under stress conditions, flux through the Krebs cycle slows and citric acid from mitochondria is released to the cytoplasm for fatty acid and fat (triacylglycerol) synthesis. Both FAD, and niacinamide used for NAD^+ synthesis, were decreased in newborns at risk for ASD (Supplementary Data 3).

Dopamine

Dopamine was decreased in newborns at risk for ASD. In 5-year-olds with ASD, dopamine was unchanged, but its metabolite, homovanillic acid (HVA) was decreased (Supplementary Data 4 and 13). Dopamine is not just a neurotransmitter. Recent studies have underscored the key importance of dopamine as a stress response molecule. Dopamine has anti-inflammatory properties [35], and improves heart, brain, and kidney functions under conditions of physiologic stress [36, 37]. Deficient production of dopamine weakens antioxidant defenses and disrupts normal dopaminergic reward circuit needed for normal social development in childhood [38]. Dopamine was not deficient in 5-year-olds with ASD (Supplementary Data 3).

Metabolic pathways associated with ASD in 5-year-old children

5-methyltetrahydrofolic acid (mTHF) and betaine (trimethyl-glycine) were decreased in males and females together, and methionine was increased only in males (Fig. 2h, Supplementary Data 4). Betaine supports mitochondrial oxidative phosphorylation by enhancing mitochondrial fusion [39] and is known to be decreased by ATP-related purinergic signaling [28]. mTHF is required for normal methionine, homocysteine, and S-adenosylmethionine (SAMe) metabolism. SAMe is needed for DNA and histone methylation, the inactivation of excitatory catecholamine neurotransmitters by the enzyme catechol-O-methyl transferase (COMT), and for many other methylation needs of differentiated cells. mTHF is the most reduced form of folate and is regulated in part by cellular redox. The $NADH/NAD^+$ and $NADPH/NADP^+$ ratios in mitochondria

and the extramitochondrial cytoplasm, respectively, help to regulate the ratios of mTHF, methylene-THF, and formyl-THF (fTHF). fTHF is the most oxidized active form of THF and relies on mitochondrial NAD^+ for synthesis. fTHF is used in *de novo* synthesis of purines like IMP, AMP, and GMP, and for formyl-methionine tRNA synthesis needed in mitochondria for protein synthesis and metabolic specialization. Methylene-THF is needed to synthesize thymidylate (dTMP) for DNA synthesis and cell growth. Disturbances of folate 1-carbon metabolism are produced by many kinds of genetic and environmental stressors that affect mitochondrial DNA replication and other mitochondrial functions [40]. While decreased in 5-year-old children with ASD, mTHF and betaine were not dysregulated in newborns at risk for ASD (Supplementary Data 3).

CoQ10 and sphingolipids

CoQ10 is a key mitochondrial electron carrier and antioxidant. CoQ10 was decreased in 5-year-olds with ASD (Fig. 2h and j). GM3(d18:1/18:1) was decreased in 5-year-old males with ASD, but not changed in the newborn cohort (Supplementary Data 3 and 4). GM3 lipids are monosialic acid containing glycosphingolipids that are used as the building blocks for all more complex gangliosides. Gangliosides are critical for cell-cell recognition and neuronal differentiation [41]. 1-deoxyceramide(m18:1/20:0) was increased in 5-year-olds with ASD (Supplementary Data 4). 1-deoxyceramdes are made by stressed cells when alanine is used instead of serine by the first committed step in sphingolipid synthesis catalyzed by the pyridoxine (B6)-dependent enzyme serine-palmitoyl transferase (SPT). 1-deoxyceramides accumulate in mitochondrial membranes, cause cytotoxic mitophagy, and trigger inflammation by activating the NLRP3 inflammasome in macrophages [42].

Endocannabinoids

Two endocannabinoids, 2-archidonylglycerol (2-AG) and N-oleoylethanolamine (N-OEA), were increased in 5-year-olds with ASD (Supplementary Data 4). 2-AG activates the peroxisome proliferator-activated receptors (PPAR) α and γ. OEA activates PPARα and β/δ. PPAR binding by 2-AG and OEA regulates lipid synthesis, glucose uptake, and inflammation [43, 44]. 2-AG also binds to the cannabinoid receptors CB1 and CB2, and OEA binds the CB2 receptor. 2-AG and OEA mediate a multifaceted pro-inflammatory response in lymphocytes and macrophages, increasing MCP-1 and IL-6 release, and nitric oxide production in macrophages and monocytes, and increasing the oxidative burst in neutrophils [45]. In addition to their peripheral pro-inflammatory effects, under conditions of neurologic stress or neuroinflammation, 2-AG and OEA are induced in the brain and are neuroprotective [46]. 2-AG and OEA can be formed from arachidonate and glycerol released from triglycerides by lipases during lipolysis that occurs during stress. Glycerol and glycerol-3-phosphate, which are precursors of lipid synthesis and products lipolysis, were elevated in 5-year-olds with ASD (Supplementary Data 4).

Antioxidant phytonutrients

Zeaxanthin was decreased in 5-year-olds with ASD (Fig. 3c, Supplementary Data 4). Zeaxanthin is a phytonutrient and carotenoid related to vitamin A that is present in the diet and has significant anti-inflammatory properties. Zeaxanthin induces NRF2 and prevents mitochondrial membrane damage caused by stress-triggered ROS [47]. Zeaxanthin was not measurable in the newborn cohort. Decreased zeaxanthin contributes to a decrease in antioxidant and anti-inflammatory capacity under conditions of physiologic or environmental stress. A related carotenoid, lutein, was decreased in the males with ASD, but lost statistical significance when males and females were merged (Supplementary Data 4).

Supplementary Discussion

A new normal—the metabolic network of ASD

A common observation in the medical management of children with ASD is that when a child has a measured deficiency in a metabolite or cofactor, that restoring normal levels is not as simple as providing a supplement. For example, we have often measured a decrease in FAD (a form of vitamin B2), nicotinamide (NAD+, vitamin B3), and methionine in children with ASD. However, when these molecules or their precursors are provided in a supplement, the excretion goes up, and the blood levels stay very nearly the same. We have interpreted this as a state of the metabolic network that defends a "new normal" in children with ASD. The network resists outside change. We reasoned that a knowledge of the TD and ASD correlation networks might provide an objective view of the new dyadic relationships between all the metabolites. Across hundreds of samples in this study, we were able to visualize and rank the positive and negative correlations between over 400 metabolites and 50 pathways. The network results reveal how changes in a metabolite, up or down even when it is still within the normal range, can affect dozens of others. For example, a prediction from the eicosanoid hub is that by changing asparagine levels by diet or supplements, the blood levels of over a dozen eicosanoids will be changed. Another implication is that the central position of purine metabolism in the networks of both newborns and 5-year-olds makes this ancient pathway uniquely positioned to have a powerful impact on the metabolic network that underlies the core symptoms of ASD.

Three layers of metabolomics

We describe three layers of the metabolome that each contribute to health and disease. In broad strokes, the metabolome of living systems contains three nested layers corresponding to *energy* (bioenergetics), *matter* (the concentration of metabolites), and *information* (the correlation state of the metabolic network and its response to stress). All of metabolism

depends on the stable foundation that generates the energy necessary for life. Mitochondria form this foundation. Upon this foundation, the matter and information functions of metabolism evolve in each tissue to create cell type specialization, and in each living organism, permitting adaptation in the face of changing environmental conditions and habitats. The results of this study showcase the changes that occur in the state of the metabolic networks during typical child development and ASD.

Decreased anti-inflammatory and antioxidant defenses

We found broad decreases in anti-inflammatory and antioxidant defenses. Markers of this response were decreased CoQ10, sphingomyelins, PC plasmalogens, dopamine, serotonin, and carnosine. Decreased capacity for cellular specialization by DNA and histone methylation and selective gene silencing was reflected by decreased 5'-methyltetrahydrofolate (mTHF) and betaine in the 5-year-old children with ASD. An early decrease in the capacity for immune regulation was marked be a decrease in isopentenyl pyrophosphate (IPP) in the newborns at risk. Later, indoxyl-3-sulfate (IS), an endogenous inhibitor of inflammation [48], was decreased in the children with ASD. There was evidence of decreased mitochondrial oxidation of the branch chain amino acid leucine in the form of decreased hydroxyisocaproic acid in newborns at risk and decreased isovalerylglycine in 5-year-old children with ASD. Decreased capacity for mitochondrial fatty acid oxidation was shown by the accumulation of long chain acyl-carnitines in newborns, and by a decrease in free carnitine in 5-year-old children with ASD. Cardiolipins needed for normal mitochondrial function were normal in the newborns at risk but decreased in 5-year-old children with ASD, further underscoring the importance of mitochondria in this stress response.

Increased stress response metabolites

There were increases in metabolic stress response molecules. These included increased citrate, glycerol, lactate, alanine and threonine, hypotaurine, cholesteryl-sulfate, pregnenolone, 7-methylguanine, and ceramides. Ceramides are made from sphingomyelins by sphingomyelinases that are activated by cell stress. Ceramides play critical roles in mitochondrial quality control and autophagy and can lead to stress-induced cell death. Glycosphingolipids were increased in both cohorts. Glycosphingolipids are key molecules used for modulating and calibrating immunity and autoimmunity [2]. Globosides and gangliosides are glycosphingolipids needed for normal tissue differentiation and brain development [49]. Although simple globosides like the MHC, DHC, and THC lipids were increased in both cohorts in this study, the ganglioside GM3 was decreased in 5-year-old males with ASD. Interestingly, gangliosides made from GM3, specifically GD2, are natural ligands for the autism-associated cellular mesenchymal-epithelial transition (c-MET) tyrosine kinase receptor [50]. Downregulation or developmental mistiming of the MET receptor can be caused by early life stress in mice [51] and was an early gene found to be dysregulated in ASD [52]. GD2 was not measured in this study, however, decreased production of GD2 would fail to trigger the normal developmental activation of c-MET and could contribute to a maturational delay. Dysregulation of endosomal trafficking, mitophagy, and autophagy was marked by decreased BMP lipids in newborns at risk. BMP lipids were increased in 5-year-old children with ASD. Bile acids have emerged as important stress-related signaling molecules [53] and play a role in neuroprotection under conditions of stress [54]. The bile acid chenodeoxycholic acid was increased in newborns and decreased in 5-year-old children with ASD.

Supplementary Methods

Metabolic network growth ($\dot{V}_{net}$) analysis

$\dot{V}_{net}$ analysis was performed with and without replacement to confirm that both methods produced comparable results (Supplementary Figures 3 and 4). The metabolic network organization of males and females contains both unique, sex-selective features, and shared features. Merging metabolomic data from males and females blurs these scientific differences. When the metabolomes of newborn females and males at future risk of ASD were combined, the resulting network connectivity, $\dot{V}_{net}$, was not correct for either single-sex network. Newborn females at risk for ASD had overconnected metabolic networks compared to female TD controls (Supplementary Fig. 5). On the other hand, newborn males at future risk of ASD had underconnected metabolic networks compared to male TD controls (Supplementary Fig. 3). There were insufficient females (8 ASD and 6 TD) in the 5-year-old cohort to adequate power a $\dot{V}_{net}$ analysis.

$\dot{V}_{net}$ analysis can be imagined as randomly grabbing n marbles from a bag of N marbles, then replacing them before shaking the bag and taking another sample. Random resampling was performed 50 times at each subsample size, from n = 4 up to N-1, where n is the subsample size and N is the cohort size (the number of blood samples analyzed). The Central Limit Theorem was then used to estimate the population mean, SD, and other statistical parameters corresponding to the number of significantly correlated metabolites at each subsample size and the q value of all possible pair-wise correlations based on the 50 random samples. This method avoided autocorrelations that are produced when a given plasma sample was correlated with itself because of replacement and chance resampling. “Sampling with replacement” methods have many more metabolite-pair correlations because of the autocorrelation artefacts that are produced. In this computational method, samples were selected one at a time from the

population, the sample noted then replaced, and the next sample selected until the desired subsample size of n samples, was achieved. After the desired n was reached, all possible pair-wise correlations were calculated. In the male cohorts, both sampling methods yielded the same results for the $\dot{V}_{net}$ ratios, although the absolute numbers for the slopes ($\dot{V}_{net}$) were higher with replacement because of the autocorrelation artefact described above.

Supplementary References

1. Valsecchi M*, et al.* APOL1 polymorphism modulates sphingolipid profile of human podocytes. *Glycoconj J* **37**, 729-744 (2020).

2. Nakayama H, Nagafuku M, Suzuki A, Iwabuchi K, Inokuchi JI. The regulatory roles of glycosphingolipid-enriched lipid rafts in immune systems. *FEBS Lett* **592**, 3921-3942 (2018).

3. Yamakawa T. A reflection on the early history of glycosphingolipids. *Glycoconj J* **13**, 123-126 (1996).

4. Bereznicka A, Mikolajczyk K, Czerwinski M, Kaczmarek R. Microbial lectome versus host glycolipidome: How pathogens exploit glycosphingolipids to invade, dupe or kill. *Front Microbiol* **13**, 958653 (2022).

5. Gudz TI, Tserng KY, Hoppel CL. Direct inhibition of mitochondrial respiratory chain complex III by cell-permeable ceramide. *The Journal of biological chemistry* **272**, 24154-24158 (1997).

6. Xin M, Deng X. Protein phosphatase 2A enhances the proapoptotic function of Bax through dephosphorylation. *The Journal of biological chemistry* **281**, 18859-18867 (2006).

7. Taniguchi M, Okazaki T. Role of ceramide/sphingomyelin (SM) balance regulated through "SM cycle" in cancer. *Cell Signal* **87**, 110119 (2021).

8. Chung H-Y, Claus RA. Keep Your Friends Close, but Your Enemies Closer: Role of Acid Sphingomyelinase During Infection and Host Response. *Frontiers in Medicine (Lausanne)* **7**, (2021).

9. Collenburg L, Schneider-Schaulies S, Avota E. The neutral sphingomyelinase 2 in T cell receptor signaling and polarity. *Biol Chem* **399**, 1147-1155 (2018).

10. Bataller M, Sanchez-Garcia A, Garcia-Mayea Y, Mir C, Rodriguez I, ME LL. The Role of Sphingolipids Metabolism in Cancer Drug Resistance. *Front Oncol* **11**, 807636 (2021).

11. Chiang YP, Li Z, Chen Y, Cao Y, Jiang XC. Sphingomyelin synthases 1 and 2 exhibit phosphatidylcholine phospholipase C activity. *The Journal of biological chemistry* **297**, 101398 (2021).

12. Barnes-Velez JA, Aksoy Yasar FB, Hu J. Myelin lipid metabolism and its role in myelination and myelin maintenance. *Innovation (Camb)* **4**, 100360 (2023).

13. Sessa L, Nardiello AM, Santoro J, Concilio S, Piotto S. Hydroxylated Fatty Acids: The Role of the Sphingomyelin Synthase and the Origin of Selectivity. *Membranes (Basel)* **11**, (2021).

14. Ou-Yang YN, Yuan MD, Yang ZM, Min Z, Jin YX, Tian ZM. Revealing the Pathogenesis of Salt-Sensitive Hypertension in Dahl Salt-Sensitive Rats through Integrated Multi-Omics Analysis. *Metabolites* **12**, (2022).

15. Neto JCR, Vieira LR, de Aquino Ribeiro JA, de Sousa CAF, Junior MTS, Abdelnur PV. Metabolic effect of drought stress on the leaves of young oil palm (Elaeis guineensis) plants using UHPLC-MS and multivariate analysis. *Sci Rep* **11**, 18271 (2021).

16. Xue LL, Chen HH, Jiang JG. Implications of glycerol metabolism for lipid production. *Prog Lipid Res* **68**, 12-25 (2017).

17. Al-Mass A*, et al.* Glycerol-3-phosphate phosphatase operates a glycerol shunt in pancreatic beta-cells that controls insulin secretion and metabolic stress. *Mol Metab* **60**, 101471 (2022).

18. Rosenfeld CS. Placental serotonin signaling, pregnancy outcomes, and regulation of fetal brain developmentdagger. *Biology of reproduction* **102**, 532-538 (2020).

19. Mao J*, et al.* Bisphenol A and bisphenol S disruptions of the mouse placenta and potential effects on the placenta-brain axis. *Proceedings of the National Academy of Sciences of the United States of America* **117**, 4642-4652 (2020).

20. Maneta E, Warren AY, Hay DP, Khan RN. Caspase-1-mediated cytokine release from gestational tissues, placental, and cord blood. *Frontiers in physiology* **6**, 186 (2015).

21. Rosenfeld CS. The placenta-brain-axis. *J Neurosci Res* **99**, 271-283 (2021).

22. Yang CJ, Tan HP, Du YJ. The developmental disruptions of serotonin signaling may involved in autism during early brain development. *Neuroscience* **267**, 1-10 (2014).

23. Lin W, Wadlington NL, Chen L, Zhuang X, Brorson JR, Kang UJ. Loss of PINK1 attenuates HIF-1alpha induction by preventing 4E-BP1-dependent switch in protein translation under hypoxia. *The Journal of neuroscience : the official journal of the Society for Neuroscience* **34**, 3079-3089 (2014).

24. Zhong Z*, et al.* New mitochondrial DNA synthesis enables NLRP3 inflammasome activation. *Nature* **560**, 198-203 (2018).

25. van der Veen JN, Kennelly JP, Wan S, Vance JE, Vance DE, Jacobs RL. The critical role of phosphatidylcholine and phosphatidylethanolamine metabolism in health and disease. *Biochim Biophys Acta Biomembr* **1859**, 1558-1572 (2017).

26. Sam PN, Avery E, Claypool SM. Proteolytic Control of Lipid Metabolism. *ACS Chem Biol* **14**, 2406-2423 (2019).

27. Gohil VM, Thompson MN, Greenberg ML. Synthetic lethal interaction of the mitochondrial phosphatidylethanolamine and cardiolipin biosynthetic pathways in Saccharomyces cerevisiae. *The Journal of biological chemistry* **280**, 35410-35416 (2005).

28. Zolkipli-Cunningham Z*, et al.* Metabolic and behavioral features of acute hyperpurinergia and the maternal immune activation mouse model of autism spectrum disorder. *PloS one* **16**, e0248771 (2021).

29. Hwang WB, Kim DJ, Oh GS, Park JH. Aryl Hydrocarbon Receptor Ligands Indoxyl 3-sulfate and Indole-3-carbinol Inhibit FMS-like Tyrosine Kinase 3 Ligand-induced Bone Marrow-derived plasmacytoid Dendritic Cell Differentiation. *Immune Netw* **18**, e35 (2018).

30. Needham BD*, et al.* Plasma and Fecal Metabolite Profiles in Autism Spectrum Disorder. *Biol Psychiatry* **89**, 451-462 (2021).

31. Elmahallawy EK*, et al.* S-Methylcysteine Ameliorates the Intestinal Damage Induced by Eimeria tenella Infection via Targeting Oxidative Stress and Inflammatory Modulators. *Front Vet Sci* **8**, 754991 (2021).

32. Riganti C, Castella B, Massaia M. ABCA1, apoA-I, and BTN3A1: A Legitimate Menage a Trois in Dendritic Cells. *Frontiers in immunology* **9**, 1246 (2018).

33. Locci A, Pinna G. Neurosteroid biosynthesis down-regulation and changes in GABA(A) receptor subunit composition: a biomarker axis in stress-induced cognitive and emotional impairment. *British journal of pharmacology* **174**, 3226-3241 (2017).

34. Bouviere J, Fortunato RS, Dupuy C, Werneck-de-Castro JP, Carvalho DP, Louzada RA. Exercise-Stimulated ROS Sensitive Signaling Pathways in Skeletal Muscle. *Antioxidants (Basel)* **10**, (2021).

35. Liu A, Ding S. Anti-inflammatory Effects of Dopamine in Lipopolysaccharide (LPS)-stimulated RAW264.7 Cells via Inhibiting NLRP3 Inflammasome Activation. *Ann Clin Lab Sci* **49**, 353-360 (2019).

36. Bucolo C, Leggio GM, Drago F, Salomone S. Dopamine outside the brain: The eye, cardiovascular system and endocrine pancreas. *Pharmacology & therapeutics* **203**, 107392 (2019).

37. Li K*, et al.* Cerebrospinal fluid and plasma metabolomics of acute endurance exercise. *FASEB journal : official publication of the Federation of American Societies for Experimental Biology* **36**, e22408 (2022).

38. Palomo R, Ozonoff S, Young GS, Belinchon Carmona M. Social orienting and initiated joint attention behaviors in 9 to 12 month old children with autism spectrum disorder: A family home movies study. *Autism research : official journal of the International Society for Autism Research* **15**, 1109-1119 (2022).

39. Jung Kim M. Betaine enhances the cellular survival via mitochondrial fusion and fission factors, MFN2 and DRP1. *Anim Cells Syst (Seoul)* **22**, 289-298 (2018).

40. Nikkanen J*, et al.* Mitochondrial DNA Replication Defects Disturb Cellular dNTP Pools and Remodel One-Carbon Metabolism. *Cell metabolism* **23**, 635-648 (2016).

41. Palmano K, Rowan A, Guillermo R, Guan J, McJarrow P. The role of gangliosides in neurodevelopment. *Nutrients* **7**, 3891-3913 (2015).

42. Lauterbach MA*, et al.* 1-Deoxysphingolipids cause autophagosome and lysosome accumulation and trigger NLRP3 inflammasome activation. *Autophagy* **17**, 1947-1961 (2021).

43. Israelian-Konaraki Z, Reaven PD. Peroxisome proliferator-activated receptor-alpha and atherosclerosis: from basic mechanisms to clinical implications. *Cardiol Rev* **13**, 240-246 (2005).

44. Fuentes E, Guzman-Jofre L, Moore-Carrasco R, Palomo I. Role of PPARs in inflammatory processes associated with metabolic syndrome (Review). *Mol Med Rep* **8**, 1611-1616 (2013).

45. Rahaman O, Ganguly D. Endocannabinoids in immune regulation and immunopathologies. *Immunology* **164**, 242-252 (2021).

46. O'Sullivan SE. An update on PPAR activation by cannabinoids. *British journal of pharmacology* **173**, 1899-1910 (2016).

47. Avila-Roman J, Garcia-Gil S, Rodriguez-Luna A, Motilva V, Talero E. Anti-Inflammatory and Anticancer Effects of Microalgal Carotenoids. *Mar Drugs* **19**, (2021).

48. Ghimire S*, et al.* Indoxyl 3-sulfate inhibits maturation and activation of human monocyte-derived dendritic cells. *Immunobiology* **223**, 239-245 (2018).

49. Cumin C, Huang YL, Everest-Dass A, Jacob F. Deciphering the Importance of Glycosphingolipids on Cellular and Molecular Mechanisms Associated with Epithelial-to-Mesenchymal Transition in Cancer. *Biomolecules* **11**, (2021).

50. Sarkar TR*, et al.* GD3 synthase regulates epithelial-mesenchymal transition and metastasis in breast cancer. *Oncogene* **34**, 2958-2967 (2015).

51. Heun-Johnson H, Levitt P. Differential impact of Met receptor gene interaction with early-life stress on neuronal morphology and behavior in mice. *Neurobiol Stress* **8**, 10-20 (2018).

52. Eagleson KL, Xie Z, Levitt P. The Pleiotropic MET Receptor Network: Circuit Development and the Neural-Medical Interface of Autism. *Biol Psychiatry* **81**, 424-433 (2017).

53. Shin DJ, Wang L. Bile Acid-Activated Receptors: A Review on FXR and Other Nuclear Receptors. *Handbook of experimental pharmacology* **256**, 51-72 (2019).

54. Weng ZB*, et al.* A Review of Bile Acid Metabolism and Signaling in Cognitive Dysfunction-Related Diseases. *Oxidative medicine and cellular longevity* **2022**, 4289383 (2022).

Supplementary Table 1. Participant characteristics. Newborn cohort.

	TD Mean ± SD (Range)	Pre-ASD[1] Mean ± SD (Range)	p value
**Subjects (n = 205)**	120	85	
**Sex**	68 male, 52 females	68 male, 17 female	n/a
**Gestational age (weeks)**	39.6 ± 1.1 (37-42)	39.5 ± 1.3 (37-42)	ns
**Birth weight (pounds)**	7.7 ± 0.7 (5-10.5)	7.6 ± 0.6 (5-10.6)	ns
**Age at sample collection (hours)**	41.8 ± 14 (18-111)	42.5 ± 17 (17-125)	ns
**IVF birth**	6.1% (7/114)	7.3% (5/69)	ns
**Gestational diabetes**	10% (11/114)	17% (12/69)	ns
**Gestational fever ≥ 101.5˚F**	8% (11/114)	9% (12/69)	ns
**Gestational prescription drugs**	0.4 ± 0.7 (0-4)	0.4 ± 0.7 (0-2)	ns
**C-section delivery (%)**	27% (31/116)	39% (27/69)	ns
**Unvaccinated (%)[2]**	5.2% (6/115)	2.9% (2/69)	ns
**Mother's age (years)**	32 ± 4 (25-42)	32 ± 4 (21-44)	ns
**Father's age (years)**	34 ± 4 (26-44)	34 ± 6 (22-50)	ns
**History of regression**	2% (2/114)	48% (33/69)	0.0001
**Age at first word (months)**	11.4 ± 1.8 (6-24)	14.7 ± 1.8 (6 to >36)	0.0001
**Age at ASD diagnosis (years)**	n/a	3.3 ± 1.1 (1-7)	n/a
**Ethnicity**			ns
**White, Caucasian**	66%	56%	
**Asian, Pacific Islander**	19%	22%	
**White, Hispanic, or Latino**	9%	17%	
**African American**	2%	1%	
**Other**	4%	5%	

[1]Newborns in the pre-ASD group developed ASD between the ages of 3 and 10 years. [2]By the age of ascertainment between 3-10 years old. **Abbreviations**: IVF, in vitro fertilization

Supplementary Table 2. Participant characteristics. 5-year-old cohort.

	TD Mean ± SD (Range)	ASD Mean ± SD (Range)	p value
**Subjects (n = 53)**	22	31	
**Sex**	16 male, 6 females	23 male, 8 female	
**Age (years)**	5.0 ± 0.7 (4.1-6.5)	5.3 ± 0.8 (4.1-7.0)	ns
**Ethnicity**[†]			ns
**African American/Black**	0	3	
**Caucasian**	12	15	
**Chinese**	0	1	
**Hispanic**[a]	0	2	
**Mixed**[b]	3	5	
**Undeclared**[c]	7	5	

*Fisher's exact test. [†]Chi-square test. [a]Includes Mexican and Brazilian. [b]Includes Hispanic/Caucasian, Chinese/Caucasian, Mexican/Caucasian, and Filipino/Caucasian. [c]Participants who chose not to identify ethnicity. **Abbreviations**: ASD, Autism Spectrum Disorder; TD, Typical Development; SD, standard deviation.

Supplementary Table 3. Metabolic network growth ($\dot{V}_{net}$) analysis.

No.	Cohort	Males	Metabolites Measured (Nodes)	Total Possible Correlations (Edges)	Significant Nodes with at least 1 significant Edge (q < 0.05)	Total Significant Edges[1] (q < 0.05)	$\dot{V}_{net}$ (mean ± sem) Global Metabolic Network Growth Rate[2] (+r and -r, edges/sample)	Positive Metabolic Network Growth Rate[2], (+r, edges/ sample)	Negative Metabolic Network Growth Rate[2] (-r, edges/ sample)
1	**Pre-ASD newborns[3]**	68	431	92,665	431	15,511****	146 ± 3.0****	121 ± 2.2****	28 ± 0.7****
2	**5-year-olds with ASD[3]**	23	467	108,811	467	3,782****	139 ± 8.7****	112 ± 6.4****	27 ± 2.4*
3	**TD newborn controls[4]**	68	431	92,665	431	18,288****	174 ± 2.1****	159 ± 1.9****	17 ± 0.4****
4	**TD 5-year-old controls[4]**	16	467	108,811	467	5,510****	315 ± 12****	275 ± 12****	38 ± 2.1*

[1]Significant correlations (edges with q < 0.05) between metabolites (nodes) that link different biochemical pathways. These are the out-of-pathway correlations. Correlations between metabolites within a given pathway, for example ATP and ADP among the purines, were not counted in this analysis. Only the networks for males were reported here. Insufficient females were enrolled to make analysis of the female networks possible.
[2]The slope of the metabolic network growth curve, $\dot{V}_{net}$, is reported as the mean ± SEM of the best fit, least squares regression line constructed from ramped random resampling analysis.
[3]ASD (autism spectrum disorder) $\dot{V}_{net}$ was the same at birth and 5-years of age.
[4]TD (typically developing) $\dot{V}_{net}$ increased by 173% in 5-year-olds compared to TD newborns. P values for the differences between the regression slope ($\dot{V}_{net}$) in ASD and age- and sex-matched TD controls were: *p < 0.05. **p < 0.01. ***p < 0.001. ****p < 0.0001.

Supplementary Fig. 1. Unique and Shared metabolic pathways disturbances in children at risk and children with ASD. a. Pre- ASD newborns. n = 205 newborns (68 males and 17 females in the pre-ASD group. 68 males and 52 females in the TD group). **b. 5-year-olds with ASD**. n = 53 5-year-olds (23 males and 8 females with ASD. 16 males and 6 females in the TD group).

a Metabolic pathway changes in newborns pre-ASD

	Fraction of impact (VIP score) explained (% of 110.4)	Molecules increased	Molecules decreased
**Metabolic pathway changtes shared in both newborns pre-ASD and in 5-yr olds with ASD**			
Phospholipid metabolism	20%	5	7
Glycosphingolipid metabolism*	10%	5	0
Ceramide metabolism*	9%	4	0
Sphingomyelin metabolism*	7%	1	3
Fatty acid oxidation and synthesis	6%	4	0
Bile salt metabolism	6%	3	0
Microbiome metabolism	5%	2	1
Purine metabolism	5%	0	3
Pyrimidine metabolism	3%	2	0
Tryptophan, kynurenine, serotonin, melatonin metabolism	2%	0	1
Histidine, histamine, carnosine metabolism	2%	0	1
Glycolysis and gluconeogenesis metabolism	1%	1	0
Amino acid metabolism (not otherwise covered)	1%	1	0
Branch chain amino acid metabolism	1%	0	1
**Metabolic pathways changed only in newborns pre-ASD**			
SAM, SAH, methionine, cysteine, glutathione metabolism	5%	2	1
Cholesterol, cortisol, non-gonadal steroid metabolism	4%	1	1
Vitamin B3 (niacin, NAD+) metabolism	2%	0	1
Vitamin B2 (riboflavin) metabolism	2%	0	1
Pentose phosphate, gluconate metabolism	2%	1	0
Krebs cycle	1%	1	0
Taurine, hypotaurine metabolism	1%	1	0
Gonadal steroids	1%	1	0
Bioamines and neurotransmitter metabolism	1%	0	1
Urea cycle	1%	0	1
**Subtotal**		**35**	**23**
**Total**		**58**	

14 of 24 pathways shared with 5-year-olds with ASD, accounting for 79% of the metabolic impact. Lipids accounted for 63% of the metabolic impact.

*Sphingolipids accounted for 26% of the metabolic impact

b Metabolic pathway changes in 5-yr-olds with ASD

	Fraction of impact (VIP score) explained (% of 111.4)	Molecules increased	Molecules decreased
**Metabolic pathway changtes shared in both newborns pre-ASD and in 5-yr olds with ASD**			
Phospholipid metabolism	26%	10	5
Sphingomyelin metabolism*	14%	1	8
Fatty acid oxidation and synthesis	5%	2	1
Glycosphingolipid metabolism*	5%	3	0
Ceramide metabolism*	5%	3	0
Pyrimidine metabolism	4%	1	2
Glycolysis and gluconeogenesis metabolism	4%	2	0
Microbiome metabolism	4%	0	2
Bile salt metabolism	3%	0	2
Tryptophan, kynurenine, serotonin, melatonin metabolism	2%	0	1
Purine metabolism	2%	1	0
Branch chain amino acid metabolism	2%	0	1
Histidine, histamine, carnosine metabolism	1%	1	0
Amino acid metabolism (not otherwise covered)	1%	1	0
**Metabolic pathways changed only in 5-yr olds with ASD**			
1-carbon, folate metabolism	3%	0	2
CoQ10 metabolism	3%	0	2
Deoxysphingolipids	3%	1	1
Endocannabinoid metabolism	3%	2	0
Amino-sugar, galactose, and non-glucose metabolism	2%	0	1
Phytonutrients, bioactive botanical metabolites	2%	0	1
Plasmalogen metabolism	2%	0	1
Cardiolipin metabolism	1%	0	1
Thyroid hormone metabolism	1%	1	0
**Subtotal**		**29**	**31**
**Total**		**60**	

14 of 23 pathways shared with newborns pre-ASD, accounting for 80% of the metabolic impact. Lipids accounted for 71% of the metabolic impact.

*Sphingolipids accounted for 24% of the metabolic impact

Supplementary Fig. 2. The rank of discriminating metabolites determined by network analysis could not be predicted by their rank measured by classical metabolomic methods, a-d. a. Mann-Whitney rank on the x-axis, **b.** Random Forest AUC-MDA rank on the x-axis, **c.** Welch's corrected p-value rank on the x-axis, **d.** Linear regression analysis**.** Multivariate AUC-VIP rank on the x-axis. Network rank on the y-axis. **Metabolite ranks calculated by classical metabolomics methods correlated with one another, e-h. e.** Random Forest MDA on the x-axis and VIP rank on the y-axis, **f.** Mann-Whitney U vs VIP, **g.** Welch's adjusted p vs VIP, **h.** Student's t test vs VIP. M = 467 measured metabolites. Sample size n = 23 5-year-old males with ASD and 16 TD control males. **Abbreviations**: AUC, area under the curve; VIP, variable importance in projection by partial least square discriminant analysis; MDA, mean decrease in accuracy.

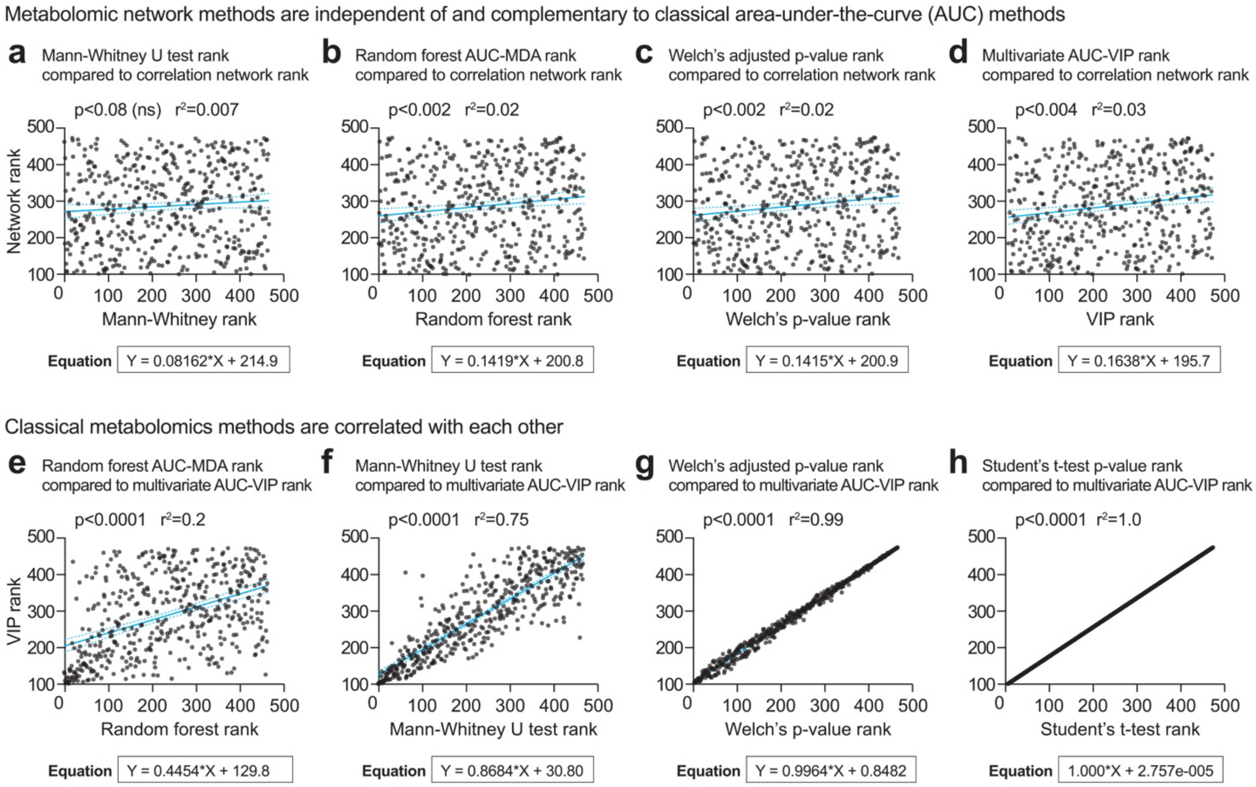

Supplementary Fig. 3. Metabolomic network growth rate ($\dot{V}_{net}$) analysis. In this example from the main text Fig. 10B, 431 metabolites (m) were measured in each of 136 metabolomes (n = 68 pre-ASD and 68 TD) in newborn males. Ramped random resampling was used to systematically estimate the number of significant network edges at each subsample size. Edges with a positive (+) and negative (-) r value were counted separately. The rate of network growth, 95% confidence intervals, and p values were calculated by least squares linear regression analysis. Regression analysis was used to derive the best fit equation for the straight line, y = mx + b. The slope of this line, m, was studied as a new parameter that quantified the rate of metabolomic network growth, $\dot{V}_{net}$, in units of edges/added sample. The ratio of (ASD $\dot{V}_{net}$)/(TD $\dot{V}_{net}$) was found to be the same when ramped random resampling was performed either with or without replacement. See Supplementary Figs. 3 and 4. Green squares: TD. Tan circles: ASD. Software for implementing this analysis is available on GitHub (https://github.com/BDNav/metabotools).

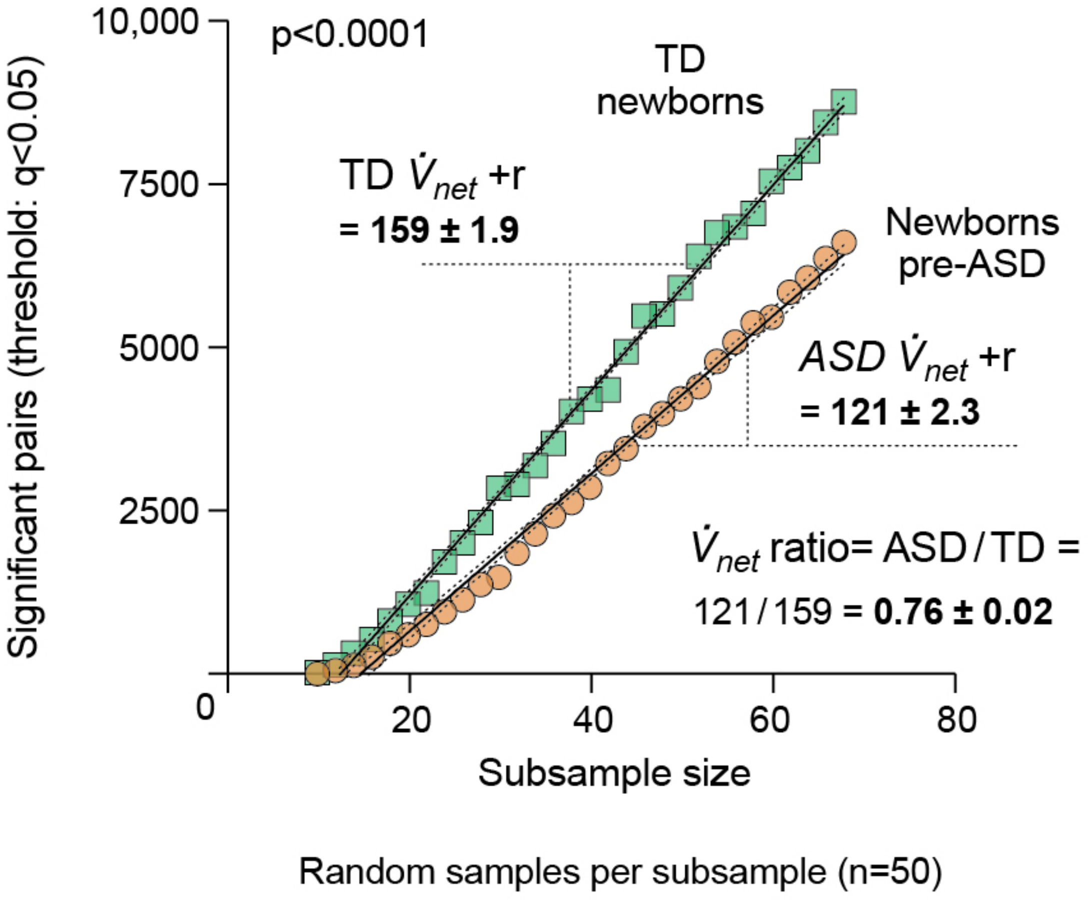

Supplementary Fig. 4. Ramped random resampling methods. $\dot{V}_{net}$ ratios, ASD/TD, in newborn males were the same when sampled with and without replacement. a-c. $\dot{V}_{net}$ calculations with replacement. a. Positive and negative (+r and -r) correlations. **b.** Positive (+r) correlations. **c.** Negative (-r) correlations. **d-f. $\dot{V}_{net}$ calculations without replacement. d**. Positive and negative (+r and -r) correlations. **e.** Positive (+r) correlations. **f.** Negative (-r) correlations.

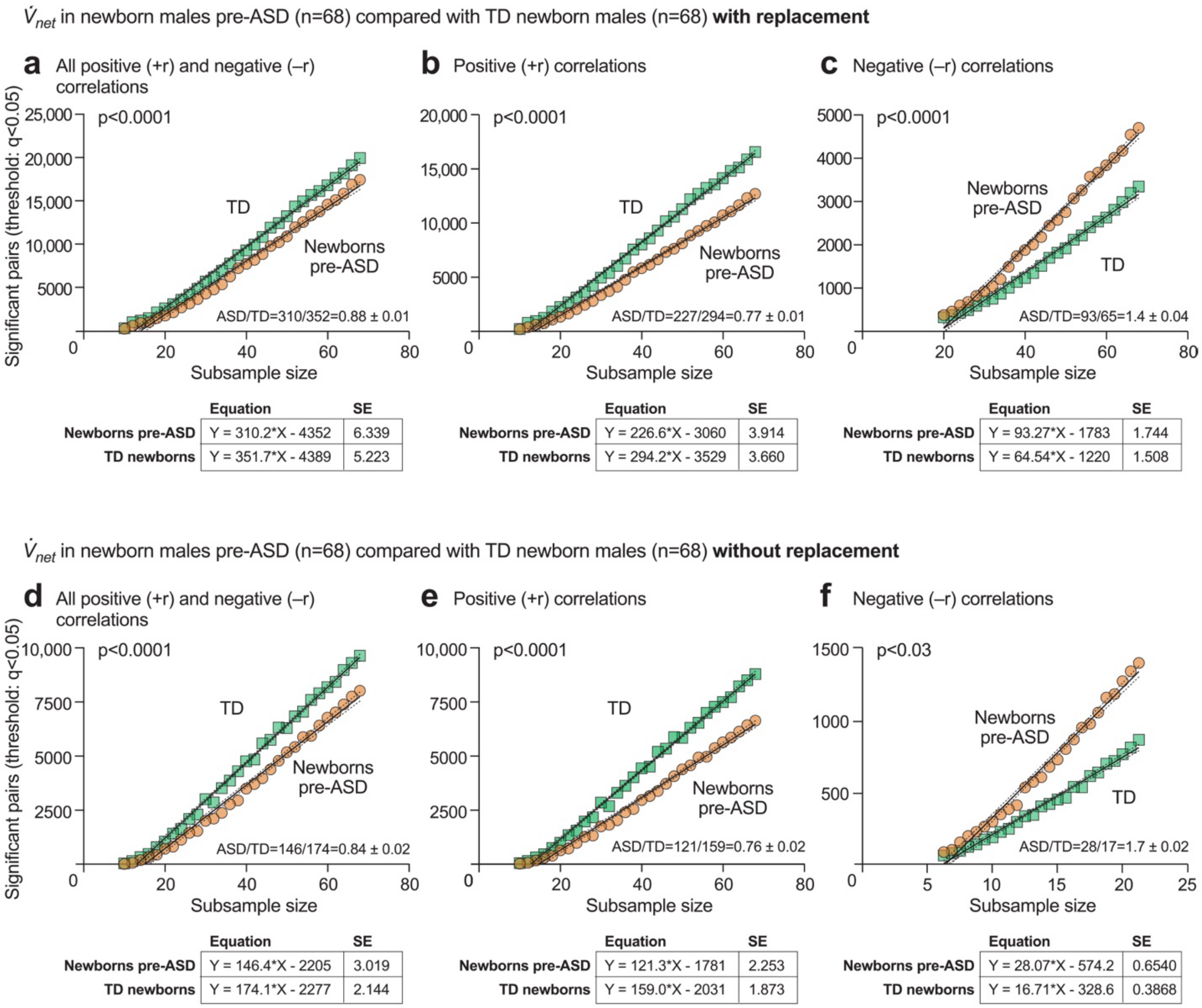

a

	Equation	SE
Newborns pre-ASD	Y = 310.2*X - 4352	6.339
TD newborns	Y = 351.7*X - 4389	5.223

b

	Equation	SE
Newborns pre-ASD	Y = 226.6*X - 3060	3.914
TD newborns	Y = 294.2*X - 3529	3.660

c

	Equation	SE
Newborns pre-ASD	Y = 93.27*X - 1783	1.744
TD newborns	Y = 64.54*X - 1220	1.508

d

	Equation	SE
Newborns pre-ASD	Y = 146.4*X - 2205	3.019
TD newborns	Y = 174.1*X - 2277	2.144

e

	Equation	SE
Newborns pre-ASD	Y = 121.3*X - 1781	2.253
TD newborns	Y = 159.0*X - 2031	1.873

f

	Equation	SE
Newborns pre-ASD	Y = 28.07*X - 574.2	0.6540
TD newborns	Y = 16.71*X - 328.6	0.3868

Supplementary Fig. 5. $\dot{V}_{net}$ ratios, ASD/TD, in 5-year-old males were the same when sampled with and without replacement. a-c. $\dot{V}_{net}$ calculations with replacement. a. Positive and negative (+r and -r) correlations. **b.** Positive (+r) correlations. **c.** Negative (-r) correlations. **d-f. $\dot{V}_{net}$ calculations without replacement.** **d**. Positive and negative (+r and -r) correlations. **e.** Positive (+r) correlations. **f.** Negative (-r) correlations.

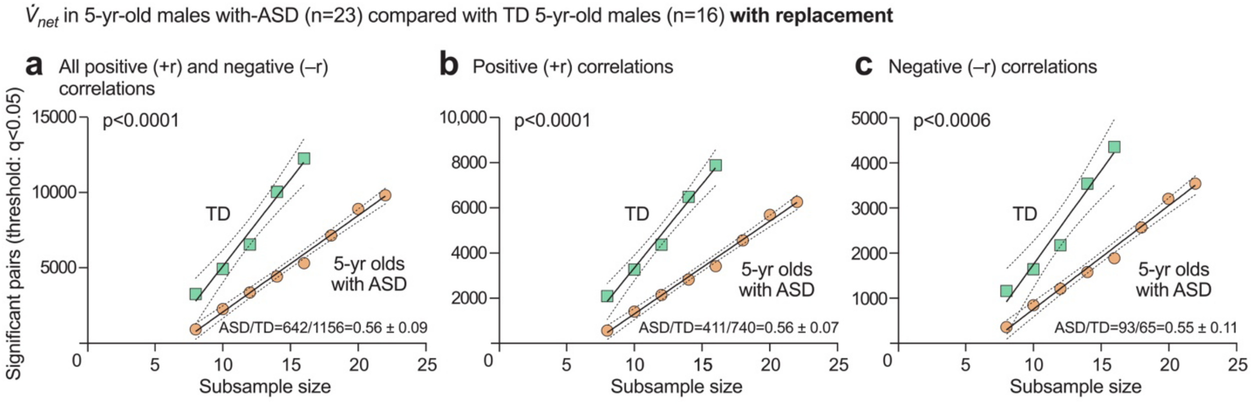

	Equation	SE
**5-yr olds with ASD**	Y = 642.0*X - 4367	25.28
**TD 5-yr olds**	Y = 1156*X - 6464	97.90

	Equation	SE
**5-yr olds with ASD**	Y = 411.2*X - 2807	15.23
**TD 5-yr olds**	Y = 740.1*X - 4060	50.98

	Equation	SE
**5-yr olds with ASD**	Y = 93.27*X - 1783	1.744
**TD 5-yr olds**	Y = 64.54*X - 1220	1.508

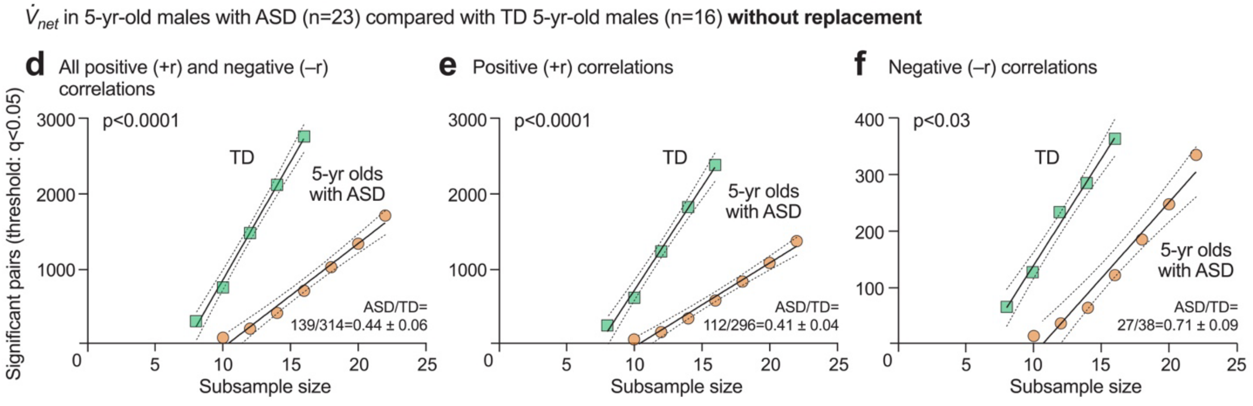

	Equation	SE
**5-yr olds with ASD**	Y = 138.6*X - 1444	8.745
**TD 5-yr olds**	Y = 314.5*X - 2299	11.97

	Equation	SE
**5-yr olds with ASD**	Y = 111.6*X - 1155	6.390
**TD 5-yr olds**	Y = 275.1*X - 2038	12.22

	Equation	SE
**5-yr olds with ASD**	Y = 26.97*X - 289.1	2.402
**TD 5-yr olds**	Y = 37.78*X - 239.1	2.107

Supplementary Fig. 6. $\dot{V}_{net}$ analysis in females was underpowered because of small sample sizes. $\dot{V}_{net}$ ratios, ASD/TD, in females were different when sampled with and without replacement. Future studies will need to enroll more females for sex-specific metabolomic and network analysis. **a-c. $\dot{V}_{net}$ calculations with replacement. a**. Positive and negative (+r and -r) correlations. **b.** Positive (+r) correlations. **c.** Negative (-r) correlations. **d-f. $\dot{V}_{net}$ calculations without replacement. d**. Positive and negative (+r and -r) correlations. **e.** Positive (+r) correlations. **f.** Negative (-r) correlations.

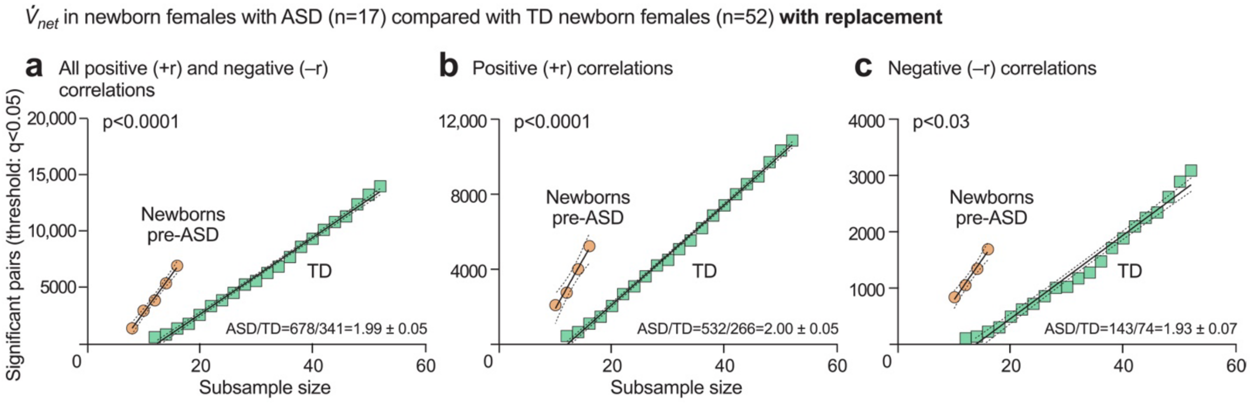

	Equation	SE
**Newborns pre-ASD**	Y = 678.0*X - 4076	33.54
**TD newborns**	Y = 340.7*X - 4245	5.724

	Equation	SE
**Newborns pre-ASD**	Y = 531.8*X - 3401	49.00
**TD newborns**	Y = 266.4*X - 3220	3.357

	Equation	SE
**Newborns pre-ASD**	Y = 143.4*X - 635.4	9.682
**TD newborns**	Y = 74.21*X - 1024	2.467

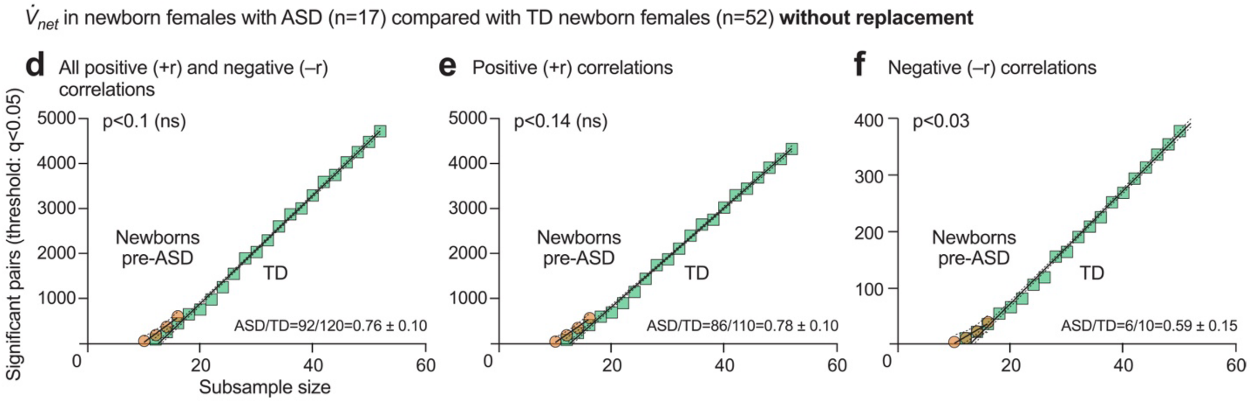

	Equation	SE
**Newborns pre-ASD**	Y = 92.13*X - 886.0	8.098
**TD newborns**	Y = 119.8*X - 1498	1.373

	Equation	SE
**Newborns pre-ASD**	Y = 86.18*X - 827.3	7.204
**TD newborns**	Y = 109.8*X - 1370	1.269

	Equation	SE
**Newborns pre-ASD**	Y = 5.870*X - 57.67	0.9330
**TD newborns**	Y = 10.01*X - 128.4	0.1452
