# Supplementary material for: Metabolic network analysis of pre-ASD newborns and 5-year-old children with autism spectrum disorder
Source: Commun Biol. 2024 May 10;7:536. doi: 10.1038/s42003-024-06102-y (PMC11549098; doi:10.1038/s42003-024-06102-y)
Supplement: Supplementary file 2 — Description of Additional Supplementary Files [file 42003_2024_6102_MOESM2_ESM.pdf]

Description of Additional Supplementary Files

File name: Supplementary Data 1
Description: Raw Metabolomic Data. Cohort #1: Pre-ASD Newborns and TD Controls. Males and females.

File name: Supplementary Data 2
Description: Raw Metabolomic Data. Cohort #2: 5-year-olds with ASD and TD Controls. Males and females.

File name: Supplementary Data 3
Description: Cohort #1: Pre-ASD Newborns and TD Controls. Metabolomic Analysis. Males and females.

File name: Supplementary Data 4
Description: Cohort #2: 5-year-olds with ASD and TD controls. Metabolomic Analysis. Males and females.

File name: Supplementary Data 5
Description: Metabolite location in the CIRCOS maps of the metabolic network in newborn males.

File name: Supplementary Data 6
Description: Metabolite location in the CIRCOS maps of the metabolic network in 5-year-old males.

File name: Supplementary Data 7
Description: Metabolic network statistics in pre-ASD newborn males. Pearson correlation analysis.

File name: Supplementary Data 8
Description: Metabolic network statistics in typically developing newborn males. Pearson correlation analysis.

File name: Supplementary Data 9
Description: Metabolic network statistics in 5-year-old males with ASD. Pearson correlation analysis.

File name: Supplementary Data 10
Description: Metabolic network statistics in typically developing 5-year-old males. Pearson correlation analysis.

File name: Supplementary Data 11
Description: Metabolic network pathway dysregulation in 5-year-old males with ASD. Ranked by pathway.

File name: Supplementary Data 12
Description: Metabolic pathway network dysregulation in pre-ASD newborn males and TD controls. Ranked by pathway.

File name: Supplementary Data 13
Description: Metabolite ranking by network. 5-year-old males with ASD and TD controls.

File name: Supplementary Data 14
Description: Metabolite ranking by network. Newborn males with ASD and TD controls.

File name: Supplementary Data 15
Description: Metabolic network growth rate (Vnet) analysis. Newborn pre-ASD males and TD controls. Positive and negative correlation tallies.

File name: Supplementary Data 16
Description: Metabolic network growth rate (Vnet) analysis. 5-year-old males with ASD and TD Controls. Positive and negative correlation tallies.

File name: Supplementary Data 17
Description: Metabolic network growth rate (Vnet) analysis. Newborn pre-ASD males and TD controls. Positive and negative correlation tallies with replacement.

File name: Supplementary Data 18
Description: Metabolic network growth rate (Vnet) analysis. 5-year-old males with ASD and TD Controls. Positive and negative correlation tallies with replacement.

File name: Supplementary Data 19
Description: Targeted metabolites and MRM data. 687 metabolites from 50 biochemical pathways were targeted in this study.
