# Supplementary material for: Metabolic network analysis of pre-ASD newborns and 5-year-old children with autism spectrum disorder
Source: Commun Biol. 2024 May 10;7:536. doi: 10.1038/s42003-024-06102-y (PMC11549098; doi:10.1038/s42003-024-06102-y)
Supplement: Supplementary file 4 — Reporting Summary [file 42003_2024_6102_MOESM4_ESM.pdf]

nature portfolio

Corresponding author(s): Robert Naviaux

Last updated by author(s): 1/29/2024

Reporting Summary

Nature Portfolio wishes to improve the reproducibility of the work that we publish. This form provides structure for consistency and transparency in reporting. For further information on Nature Portfolio policies, see our Editorial Policies and the Editorial Policy Checklist.

Statistics

For all statistical analyses, confirm that the following items are present in the figure legend, table legend, main text, or Methods section.

n/a	Confirmed	
☐	☒	The exact sample size (*n*) for each experimental group/condition, given as a discrete number and unit of measurement
☐	☒	A statement on whether measurements were taken from distinct samples or whether the same sample was measured repeatedly
☐	☒	The statistical test(s) used AND whether they are one- or two-sided<br>*Only common tests should be described solely by name; describe more complex techniques in the Methods section.*
☐	☒	A description of all covariates tested
☐	☒	A description of any assumptions or corrections, such as tests of normality and adjustment for multiple comparisons
☐	☒	A full description of the statistical parameters including central tendency (e.g. means) or other basic estimates (e.g. regression coefficient) AND variation (e.g. standard deviation) or associated estimates of uncertainty (e.g. confidence intervals)
☐	☒	For null hypothesis testing, the test statistic (e.g. *F*, *t*, *r*) with confidence intervals, effect sizes, degrees of freedom and *P* value noted<br>*Give P values as exact values whenever suitable.*
☒	☐	For Bayesian analysis, information on the choice of priors and Markov chain Monte Carlo settings
☒	☐	For hierarchical and complex designs, identification of the appropriate level for tests and full reporting of outcomes
☐	☒	Estimates of effect sizes (e.g. Cohen's *d*, Pearson's *r*), indicating how they were calculated

Our web collection on statistics for biologists contains articles on many of the points above.

Software and code

Policy information about availability of computer code

Data collection	Mass spectrometry data were initially captured and processed using the commercially available programs MultiQuant (Sciex LC-MS/MS data) and MassHunter (Agilent GC-MS/MS).
Data analysis	Statistical analysis and data visualization were performed using MetaboAnalyst 5.0 (https://www.metaboanalyst.ca) and GraphPad Prism (https://www.graphpad.com). Additional code for analysis is available at GitHub (https://github.com/BDNav/metabotools).

For manuscripts utilizing custom algorithms or software that are central to the research but not yet described in published literature, software must be made available to editors and reviewers. We strongly encourage code deposition in a community repository (e.g. GitHub). See the Nature Portfolio guidelines for submitting code & software for further information.

Data

Policy information about availability of data

All manuscripts must include a data availability statement. This statement should provide the following information, where applicable:

- Accession codes, unique identifiers, or web links for publicly available datasets
- A description of any restrictions on data availability
- For clinical datasets or third party data, please ensure that the statement adheres to our policy

Raw mass spectrometry data is available in Supplementary Tables S1 and S2.

Research involving human participants, their data, or biological material

Policy information about studies with human participants or human data. See also policy information about sex, gender (identity/presentation), and sexual orientation and race, ethnicity and racism.

Reporting on sex and gender	Data from males and females were analyzed in this study. The word gender was not used.
Reporting on race, ethnicity, or other socially relevant groupings	Commonly used categories of ethnicity were used as descriptors in the demographic Tables 1 and 2. No group-specific analysis was performed on the basis of ethnicity.
Population characteristics	Newborn children and 5-year-old children of both sexes were enrolled in this study. The study design was case-control.
Recruitment	Children born in California, ages 3 years to 10 years, with the diagnosis of autism spectrum disorder, and typically developing controls, were recruited by word of mouth and an online study description for the newborn screening study, and parents asked for permission to analyze the dried blood spots that were collected at birth as part of the universal newborn screening program. Children in the 5-year-old cohort were originally recruited as part of the CHARGE (CHildhood Autism Risks from Genetics and Environment) Study. Samples were collected prior to routine, pre-kindergarten, well-child visits.
Ethics oversight	This study was reviewed and approved by the Office for Human Research Protections (OHRP) and Institutional Review Boards (IRBs) at the University of California, San Diego, University of California, Davis, and the State of California Committee for the Protection of Human Subjects.

Note that full information on the approval of the study protocol must also be provided in the manuscript.

Field-specific reporting

Please select the one below that is the best fit for your research. If you are not sure, read the appropriate sections before making your selection.

☒ Life sciences ☐ Behavioural & social sciences ☐ Ecological, evolutionary & environmental sciences

For a reference copy of the document with all sections, see nature.com/documents/nr-reporting-summary-flat.pdf

Life sciences study design

All studies must disclose on these points even when the disclosure is negative.

Sample size	205 samples were collected in the newborn cohort. 53 samples were collected in the 5-year-old cohort. Previous metabolomics studies showed that single-sex group sizes of >15-20 provided adequate statistical power.
Data exclusions	No participant was excluded on the basis of sex, ethnicity, or genetics. After sample analysis and quality control checks, no sample results were excluded.
Replication	Metabolomics permutation analysis, repeated double cross validations (rdCVs), and area under the receiver operator characteristic (AUROC) curve analyses were performed to confirm the univariate and multivariate results reported. Metabolomics network correlation analysis was performed using bootstrap and random resampling methods that permitted mean edge counts, SD, false discovery rates (FDRs), Bayesian q values, p-values, and 95% confidence intervals to calculated for each network analyzed.
Randomization	Children allocated into the ASD group met DSMIV criteria for autistic disorder. Age- and sex-matched children without autistic disorder, and without a known chronic illness were enrolled as typically developing controls.
Blinding	This was a non-intervention study.

Reporting for specific materials, systems and methods

We require information from authors about some types of materials, experimental systems and methods used in many studies. Here, indicate whether each material, system or method listed is relevant to your study. If you are not sure if a list item applies to your research, read the appropriate section before selecting a response.

Materials & experimental systems

n/a	Involved in the study
☒	☐ Antibodies
☒	☐ Eukaryotic cell lines
☒	☐ Palaeontology and archaeology
☒	☐ Animals and other organisms
☐	☒ Clinical data
☒	☐ Dual use research of concern
☒	☐ Plants

Methods

n/a	Involved in the study
☒	☐ ChIP-seq
☒	☐ Flow cytometry
☒	☐ MRI-based neuroimaging

Clinical data

Policy information about clinical studies

All manuscripts should comply with the ICMJE guidelines for publication of clinical research and a completed CONSORT checklist must be included with all submissions.

Clinical trial registration

n/a

Study protocol

This study was approved by the University of California, San Diego (#140072, #171940, #190972), California state (#1162, #2018-020), and University of California, Davis (#226004-4) institutional review boards. Informed signed consent was obtained prior to participation.

Data collection

Plasma samples for the study of 5-year-olds were collected 8/25/2010 to 1/9/2013. Dried blood spots were collected from newborn children as part of the California universal newborn screening program from 11/23/2007 to 1/29/2018.

Outcomes

This was not an intervention study. There were no clinical outcomes to be collected.
